# βC1 protein encoded in geminivirus satellite concertedly targets MKK2 and MPK4 to counter host defense

**DOI:** 10.1371/journal.ppat.1007728

**Published:** 2019-04-18

**Authors:** Tao Hu, Changjun Huang, Yuting He, Claudia Castillo-González, Xiaojian Gui, Yaqin Wang, Xiuren Zhang, Xueping Zhou

**Affiliations:** 1 State Key Laboratory of Rice Biology, Institute of Biotechnology, Zhejiang University, Hangzhou, China; 2 State Key Laboratory for Biology of Plant Diseases and Insect Pests, Institute of Plant Protection, Chinese Academy of Agricultural Sciences, Beijing, China; 3 Department of Biochemistry and Biophysics, Texas A&M University, College Station, TX, United States of America; 4 Institute for Plant Genomics and Biotechnology, Texas A&M University, College Station, TX, United States of America; University of California, Davis Genome Center, UNITED STATES

## Abstract

Plant viruses have evolved multiple strategies to overcome host defense to establish an infection. Here, we identified two components of a host mitogen-activated protein kinase (MAPK) cascade, MKK2 and MPK4, as *bona fide* targets of the βC1 protein encoded by the betasatellite of tomato yellow leaf curl China virus (TYLCCNV). βC1 interacts with the kinase domain of MKK2 and inhibits its activity. *In vivo*, βC1 suppresses flagellin-induced MAPK activation and downstream responses by targeting MKK2. Furthermore, βC1 also interacts with MPK4 and inhibits its kinase activity. TYLCCNV infection induces the activation of the MAPK cascade, mutation in *MKK2* or *MPK4* renders the plant more susceptible to TYLCCNV, and can complement the lack of βC1. This work shows for the first time that a plant virus both activates and suppresses a MAPK cascade, and the discovery of the ability of βC1 to selectively interfere with the host MAPK activation illustrates a novel virulence function and counter-host defense mechanism of geminiviruses.

## Author summary

We identified a plant MAPK cascade that contributes to the defense against geminiviruses. Rapid activation of MAPKs was observed upon geminivirus infection. The βC1 protein of geminivirus betasatellite interacts with MKK2 and MPK4 and inhibits their kinase activity. Also, βC1 suppresses flagellin-induced MAPK activation and downstream responses. Knocking out two components of this cascade, MKK2 or MPK4 in tobacco renders the plant more susceptible to geminivirus infection and virus infection of geminivirus without betasatellite induced typical virus symptom in MKK2 or MPK4 knockout tobacco, indicating that MKK2 or MPK4 mutant can complement the lack of βC1 in virus infection. This study unveils MAPK cascades as novel players in anti-viral defense and illustrates how plant viruses have in turn evolved to suppress this pathway.

## Introduction

Plants have evolved multiple layers of effective strategies to defend themselves from invading pathogens [1]. The first layer of immune response depends on the recognition of pathogen- or microbe-associated molecular patterns (PAMPs or MAMPs) by cell membrane-associated pattern recognition receptors (PRRs). Perception of PAMPs/MAMPs by PRRs activates a signaling cascade that ultimately results in PAMP-triggered immunity (PTI) [2], which is fast and transient. Multiple PTI readouts are produced downstream of PRR activation, including activation of cascades of Mitogen-Activated Protein Kinases (MAPK) and the subsequent up-regulation of defense gene expression. On the other hand, pathogens can produce virulence factors, such as bacterial effectors, to counter PTI [3]. The second layer of immune response is triggered by the recognition of pathogen effectors in a direct or indirect manner, and is called effector triggered immunity (ETI) [1]. ETI is normally stronger and more persistent than PTI, and is often manifested in the hypersensitive response (HR), which restricts pathogen growth [1]. ETI can also activate MAPKs and defense gene expression [4,5]. For instance, in *Arabidopsis thaliana*, MAPK cascades can be rapidly activated by the recognition of PAMPs/MAMPs by their cognate PRRs, as has been demonstrated for bacterial flagellin, bacterial EF-Tu, or fungal chitin [6–8], and at least one bacterial effector, namely AvrRpt2, has been shown to strongly activate two of the *Arabidopsis* MAPKs, AtMPK3 and AtMPK6 [9].

In *A*. *thaliana*, a typical MAPK cascade is fulfilled through three sequential kinase reactions that involve MAPK kinase kinases (MEKKs), MAPK kinases (MKKs), and MAPKs [4–5,10]. Different combinations of the kinases above result in two MAPK branches: one is through MEKK1-MKK1/MKK2-MPK4 [11–13], whereas the other is through MEKKs-MKK4/MKK5-MPK3/MPK6 [14–16]. These two branches participate in plant immunity though regulating phytohormone signaling, expression of a subset of defense-related genes, and synthesis of antimicrobial compounds [4, 10]. For instance, activated MPK3/MPK6 can phosphorylate 1-aminocyclopropane-1-carboxylic acid synthase (ACS) isoforms, ACS2 and ACS6, and stabilize these proteins, leading to the induction of ethylene biosynthesis [17, 18]. In contrast, the MPK4 cascade was originally considered as a negative regulator of defense [11–13]. Both mpk4 knockout mutant and mkk1 mkk2 double knockouts in *Arabidopsis* exhibit autoimmune responses such as elevated salicylic acid (SA) and reactive oxygen species (ROS) accumulation, constitutive expression of PATHOGENESIS-RELATED GENE1 (*PR1*) and *PR2*, insensitivity to jasmonic acid (JA), and increased resistance to the plant pathogenic bacteria *Pseudomonas syringae* DC3000 [11–12, 19]. Conversely, *Arabidopsis* plants expressing constitutively active MPK4 show enhanced susceptibility to pathogens [20]. MPK4 was found to phosphorylate the transcriptional regulators ARABIDOPSIS SH4-RELATED (ASR3) and MPK4 SUBSTRATE1 (MKS1) to repress the expression of a subset of defense-related genes [21–23]. Strikingly, this cascade is also reported to positively regulate defense: about 50% of the flg22 (conserved epitope derived from bacterial flagellin)-induced genes require MPK4 for regulation [24], and transgenic *Arabidopsis* plants expressing constitutively active MKK2 show increased resistance against *P*. *syringae* and *Erwinia carotovora* [25]. The autoimmune responses caused by *mpk4* and *mkk1/mkk2* mutants can be suppressed by mutation in *SUMM2*, which encodes a nucleotide binding leucine-rich repeat (NB-LRR) protein, and *summ2/mkk1/mkk2* triple mutants exhibit enhanced susceptibility to *P*. *syringae* DC3000 and *Hyaloperonospora arabidopsidis* (*H*.*a*.) Noco2. These findings indicate that the MKK2/MPK4 cascade is also involved in mediating host defense [26, 27].

Pathogens have evolved different tactics to interfere with plant immunity in order to achieve a successful infection [4, 10]; among these, the MAPK cascade has been described as a common target of bacterial effectors [28]. For example, it has been shown that *P*. *syringae* type III effector HopF2 can directly inactivate the MKK5 activity by depositing ADP-ribose moieties to the protein [29]. Another case is that of the *P*. *syringae* effector HopAI1. This protein possesses unique phosphorylated threonine lyase activity and catalyzes the removal of the phosphate group from phosphothreonine residues of MAPKs, leading to the suppression of defense-related genes and a decrease in callose deposition [30]. In addition, flagellin-induced MPK4 and MPK11 activation is specifically suppressed by AvrRpt2 [31]. Taken together, these results suggest that interference with the MAPK cascades appears to be a widely used tactic for bacterial pathogens to counter host defense. Also, silencing the orthologss of MPK3 and MPK6 in tobacco MAPK pathway (WIPK and SIPK) attenuated N gene-mediated resistance against tobacco mosaic virus (TMV) [32], indicating that MAPK cascades may also play an important role in anti-viral defense.

*Begomovirus* is a genus in the family *Geminiviridae*. Begomoviruses contain single-stranded DNA genomes, infect economically important crops and cause serious damages in agriculture worldwide [33]. Begomoviruses can be further classified into monopartite and bipartite subgroups according to the number of their genome components. Functional betasatellites have been found to be associated with monopartite begomoviruses [34, 35]. Betasatellites are circular single-stranded DNA molecules of approximately 1,350 nucleotides, and only encode one protein, named βC1, which has been found to dramatically enhance the symptoms of the helper viruses [36, 37]. Tomato yellow leaf curl virus betasatellite (TYLCCNB)-encoded βC1 protein has been shown to change leaf polarity by binding to ASYMMETRIC LEAVES 1 (AS1) in *Arabidopsis*, and thus alters leaf development by reducing miR165/166 levels and increasing *PHABULOSA (PHB)* and *PHAVOLUTA (PHV)* transcript levels [36]. In addition, Cotton leaf curl Multan virus betasatellite (CLCuMuB)-encoded βC1 protein can subvert plant ubiquitination through interacting with S-phase kinase-associated protein 1 (SKP1) to impair the integrity of the SKP1/Cullin1 (CUL1)/F-box (SCF) complex SCF^COI1^, and thus promotes virus infection and symptom induction [38]. Besides inducing typical viral symptoms, βC1 can also suppress multiple host defense pathways, including transcriptional gene silencing (TGS) [39], post-transcriptional gene silencing (PTGS) [40–42], and JA signaling [36, 38, 43]. The mechanisms underlying the development of most symptoms determined by βC1 (e.g. vein thickening and presence of enations) remain unclear, and it is hypothesized that βC1 might also impair other host developmental or defense pathways.

In this study, we show that geminivirus infection activates MAPK signaling in *Nicotiana benthamiana* and *A*. *thaliana*, thus extending the defense role of the MAPK pathway to plant viruses. In addition, our results demonstrate that geminivirus-encoded βC1 concurrently targets two components of the MAPK signaling cascade, MKK2 and its substrate MPK4, to suppress host defense and promote infection. These findings provide new insight on the molecular arms race between plants and viruses, uncovering MAPK cascades as players in anti-viral defense, and illustrating how plant viruses have in turn evolved to suppress this pathway.

## Results

### Identification of MKK2 as a *bona fide* target of βC1

**Fig 1:**
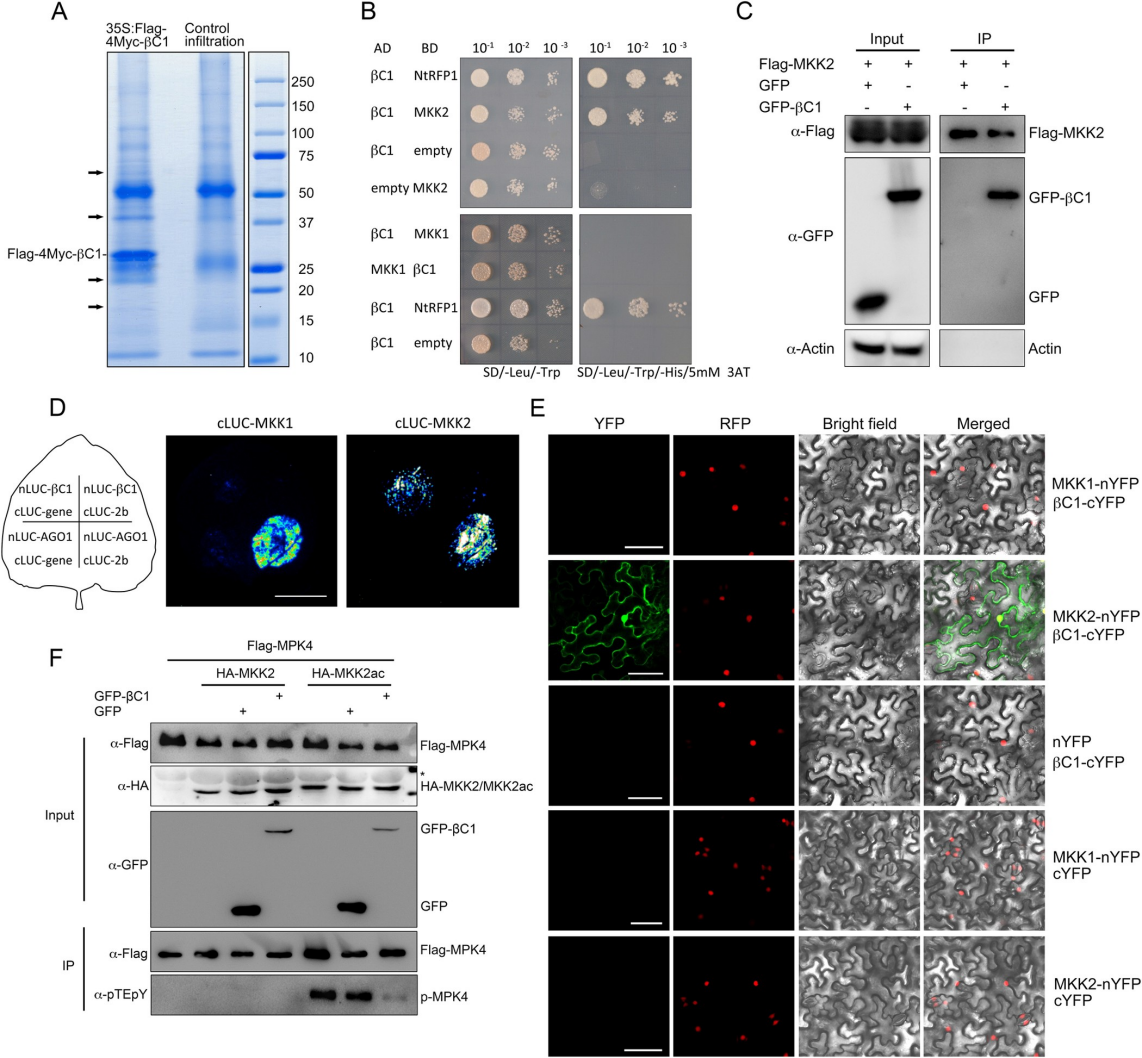
Identification of MKK2 as a new *bona fide* target of TYLCCNB βC1. (**A**) Commassie Brilliant Blue stained SDS-PAGE of immunoprecipiated βC1 complexes prepared from *Nicotiana benthamiana* for proteomics analysis. Arrows indicate collected bands. (**B**)**-**(**E**) Specific βC1-MKK2 interaction was confirmed by Y2H (**B**), Co-IP (**C**), LCI (**D**), and BiFC (**E**) assays. In (**B**), Yeast strain Gold transformed with the indicated plasmid combinations was spotted with 10-fold serial dilutions on synthetic dextrose (SD)/-Leu/-Trp medium and SD/-Leu/-Trp/-His containing 5 mM 3-aminotriazole (3-AT). Interaction between βC1 and NtRFP1 serves as the positive control. In (**C**), *N*. *benthamiana* leaves were infiltrated with *A*. *tumefaciens* cells harboring 3Flag-MKK2 with GFP-βC1 or GFP. Co-IP assay was carried out with anti-Flag M2 magnetic beads (Sigma). Samples before (Input) and after (IP) immunopurification were analyzed by immunoblot using anti-GFP and anti-Flag antibody, actin serves as a control. In left panel of (**D**), Schematic diagram shows the combinations of the infiltrated constructs used for the luciferase complementation assays. In the right panel, the fluorescence signal at different parts of the leaf shows the protein-protein interaction. The combination of NLuc-AGO1 with CLuc-CMV 2b serves as the positive control. In (**E**), MKK2-nYFP and βC1-cYFP were transiently expressed in the *35S-RFP-H2B* transgenic *N*. *benthamiana* leaves. Combinations of agro-infiltrated constructs were indicated. Columns from left to right represent YFP fluorescence, RFP fluorescence, bright field and YFP/RFP/bright field overlay. Bars represent 50 μm. (**F**) Immunocomplex kinase assay shows that βC1 reduces MKK2 mediated MPK4 activation. Flag-MPK4 was transiently expressed in *N*. *benthamiana* leaves with HA-tagged MKK2 or its constitutively active form (HA-MKK2/HA-MKK2ac) and GFP-tagged βC1 (GFP-βC1). Flag-MPK4 was immunoprecipitated by α-Flag conjugated beads and subjected for immunoblot using anti-pTEpY antibody. The protein loading of Flag-MPK4, HA-MKK2/MKK2ac, GFP-βC1 and GFP are shown by immunoblot. The asterisk (*) indicates cross-reaction band. Three biological replicates were performed.

To identify the cellular factors targeted by βC1 protein, we pursued a proteomics approach. Briefly, we transiently expressed *Flag-4Myc(FM)-βC1* in *N*. *benthamiana* leaves, and isolated βC1-containing complexes through a two-step immunoprecipitation (IP) [44]. We resolved the IPed products in 4–20% SDS-PAGE gradient gels (Fig 1A). Distinct bands that were visible in the βC1 IP but absent from the control IP were excised from the gel and analyzed by mass spectrometry (MS). In one band, we recovered seven unique peptides that matched a *N*. *benthamiana* MKK protein (encoded by Niben101scf02790g03012.1) (S1A Fig). This protein, of about 40 kDa, is the ortholog of *N*. *tabacum* salicylic acid-induced protein kinase kinase (NtSIPKK) [45] and *Arabidopsis* mitogen-activated protein kinase kinase 2 (MKK2), with 99% and 68% amino acid similarity, respectively (S1B Fig). We named this protein NbSIPKK and decided to use *Arabidopsis* MKK2 as a surrogate for further studies.

To validate the interaction between βC1 and MKK2, we first performed a yeast two-hybrid assay (Y2H). Full-length open reading frames (ORFs) encoding MKK2 and βC1 were fused to the GAL4 DNA-binding and activation domains, respectively. Yeast transformants harboring both BD-MKK2 and AD-βC1 were readily grown in the selection medium, same as the positive control AD-βC1 and BD-*Nicotiana tabacum* RING FINGER PROTEIN 1 (NtRFP1) [46], revealing that βC1 interacts physically with MKK2 of *Arabidopsis* (Fig 1B). We also conducted a Co-immunoprecipitation (Co-IP) assay upon transient expression in *N*. *benthamiana* leaves. Flag-MKK2 was co-expressed with GFP-βC1 or GFP in *N*. *benthamiana* leaves and then subjected to immunoprecipitation using an anti-Flag antibody, and Co-IP products were detected with an anti-GFP antibody. Again, a specific interaction between MKK2 and βC1, but not between MKK2 and GFP protein or endogenous actin protein, was observed in the Co-IP assay (Fig 1C). We next performed a firefly luciferase complementation imaging (LCI) assay. For this assay, βC1 and MKK2 proteins were fused to the N- and C-terminal parts of firefly luciferase protein (NLuc and CLuc), respectively. The fusion proteins were transiently expressed in *N*. *benthamiana* leaves. The combination of βC1 and MKK2, similar to the positive control, AGO1-CMV2b [47], clearly restored the catalytic activity of luciferase (Fig 1D), further confirming the interaction of these two proteins *in planta*. Notably, MKK2 has a paralog in *Arabidopsis*, MKK1, with 66% amino acid similarity, and it is believed that these two proteins function redundantly in the MEKK1-MKK1/MKK2-MPK4 signaling cascade [11–13]. However, we did not detect an interaction between βC1 and MKK1 in Y2H nor LCI assays (Fig 1B and 1D). Besides, the interaction between NbSIPKK and βC1 was also confirmed by the Co-IP assay (S1C Fig). These results suggest that βC1 interacts with MKK2 specifically.

To investigate the subcellular localization of the βC1-MKK2 interaction *in planta*, we carried out a bimolecular fluorescence complementation (BiFC) assay. To this end, βC1 and MKK2 were fused to split N- or C- terminal parts of the yellow fluorescent protein (YFP), respectively, and expressed in transgenic *N*. *benthamiana* harboring *red fluorescent protein (RFP)-H2B*. Confocal microscopy detected the presence of the YFP fluorescence in both cytoplasm and nucleus (Fig 1E and S1D Fig), indicating that βC1 and MKK2 interact in these two subcellular compartments. This is in agreement with the subcellular localization described for these two proteins [12, 28]. Consistent with the results from Y2H and LCI assays, BiFC failed to detect an interaction between βC1 and MKK1 (Fig 1E).

To map the interaction interface between MKK2 and βC1, we generated three truncated versions of MKK2, namely N-terminal part (NTP, amino acid 1–157), C-terminal part (CTP, amino acid 157–372) and Serine/Threonine kinase catalytic domain (KD, amino acid 79–339; Database: Smart; Accession: SM00220) (S2A Fig). BiFC assays showed that βC1 interacted with MKK2-CTP and MKK2-KD (S2B and S2C Fig), suggesting that the βC1 protein interacts specifically with the kinase domain of MKK2.

### βC1 inhibits MKK2 kinase activity

The specific interaction of βC1 with the MKK2 kinase domain prompted the question of whether βC1 affected MKK2 kinase activity. To test this, we conducted an immunocomplex kinase assay in *N*. *benthamiana* leaves. We expressed HA epitope-tagged MKK2 or the constitutively active form of MKK2 (MKK2ac) with its *bona fide* substrate [14], Flag-tagged mitogen-activated protein kinase 4 (MPK4) and GFP-βC1 or GFP in *N*. *benthamiana* leaves by agroinfiltration. Flag-MPK4 was immunoprecipiated, and phosphorylated Flag-MPK4 protein was then detected by immunoblot using anti-pTEpY antibody [21]. The constitutively active form of MKK2 activates MPK4 in *N*. *benthamiana* leaves, and GFP-βC1, but not GFP alone, significantly reduced the phosphorylation of Flag-MPK4 by MKK2ac (Fig 1F). This indicates that βC1 inhibits the kinase activity of MKK2 on MPK4.

### Geminivirus infection induces the MAPK defense pathway

**Fig 2:**
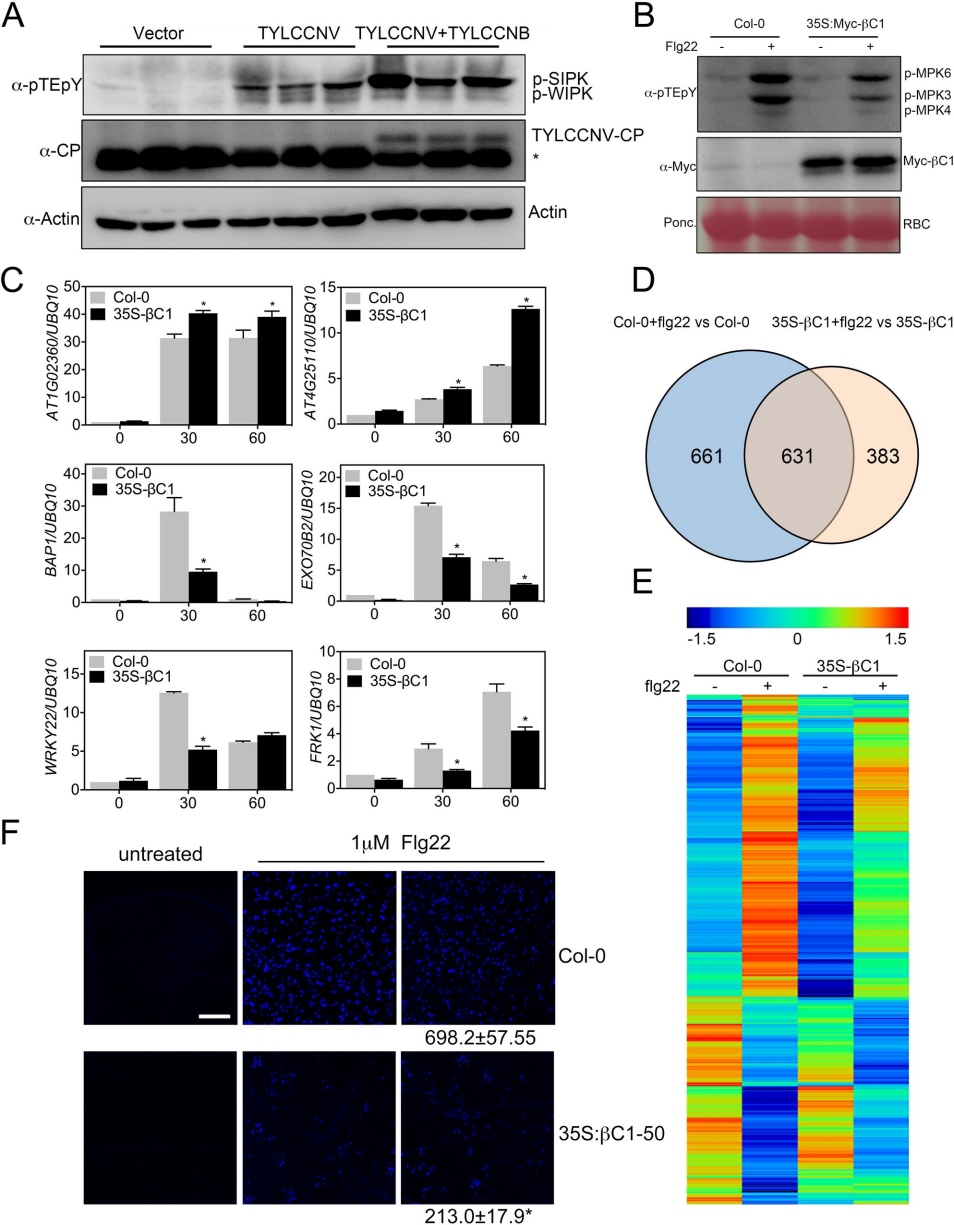
βC1 inhibits the MAPK cascade that is activated by virus infection. (**A**) MAPK activation in mock infiltrated and virus-infiltrated *N*. *benthamiana*. Seven-leaf-stage *N*. *benthamiana* plants were infiltrated with Agrobacterium harboring empty vector, TYLCCNV and TYLCCNV with TYLCCNB. Systemic leaves were collected seven days post infiltration. Endogenous phosphorylated NbSIPK and NbWIPK were monitored by immunoblot with an anti-pTEpY antibody. Virus accumulation was detected by a TYLCCNV-CP antibody. Actin protein serves as a loading control. The asterisk (*) indicates cross-reaction band. (**B**) Flg22-induced MAPK activation in Col-0 and *35S-Myc-βC1*. 10-day seedlings were treated with 100 nM flg22 for 15 min and subjected to immunoblot assays with an anti-pTEpY or anti-Myc antibody. Ponceau S staining of Rubisco (RBC) shows protein loading. Numbers indicate the average ratio of phosphorylated MPK6 and MPK3 protein in *35S-Myc-βC1* transgenic plants compared wild type for four biological replicates, asterisks represent statistically significant based on Student’s t test at P < 0.05. (**C**) RT-qPCR analysis of Flg22-induced expression of selected marker genes in the MAPK pathways in Col-0 and *35S-Myc-βC1*. Ten-day-old seedlings were treated with 1μM flg22 for 30 and 60 min. The asterisk (*) indicates significant differences with a Student’s t test (P < 0.05). (**D**) Venn diagram of flg22-regulated genes (fold change ≥2 and P value < 0.05) in Col-0 and *35S-Myc-βC1* (100 nM flg22 treatment for 30 min). (**E**) Heat map of flg22-regulated genes in Col-0 and *35S-Myc-βC1* transgenic plants with or without flg22 treatment. Hierarchical clustering was analyzed with the average linkage method using MeV. Red color to blue color indicates expression level from relatively high to low. (**F**) βC1 attenuates flg22 induced callose deposition. Flg22-induced callose deposition in Col-0 and *35S-Myc-βC1* transgenic plants. Ten-day-old Arabidopsis seedlings were treated with 1μM flg22 for 24h. Callose deposition was analyzed after 4-hour Aniline blue staining. Bars represent 0.1 mm. The number below each microscopy photograph indicates the average number and standard error of mean of callose deposits. The results are a representative of five independent experiments. Asterisk represents statistically significant based on Student’s t test at P < 0.05.

MAPK cascades coincide with early host defense responses to invading pathogens such as *Alternaria brassicicola* (*A*. *brassicicola*) and *P*. *syringae* [8, 14]. Given that βC1 interacts with MKK2 *in vivo* and suppresses its kinase activity, we hypothesized that the MAPK pathway might be a defense response against geminivirus infection. To test this idea, we inoculated *N*. *benthamiana* plants with Tomato yellow leaf curl China virus (TYLCCNV) and TYLCCNV with betasatellite (TYLCCNB) and checked the phosphorylation status of the MAPK pathway using NbSIPK (the *N*. *benthamiana* ortholog of AtMPK6) as a proxy. We first detected viral load in systemic infected leaves by western blot against the viral coat protein and qPCR assay, and the amount of CP and βC1 mRNA was detected by RT-PCR (S3A and S3B Fig). Western blot analysis showed that the virus infection indeed significantly increased the levels of NbSIPK phosphorylation in systemic leaves at seven days post inoculation (Fig 2A) [48]. Tobacco mosaic virus induces the activation of WIPK (orthologous of AtMPK3) at 30 hours after inoculation, and the increased amount of WIPK mRNA could still be detected in systemic leaves 12 days post virus infection [49]. Purified TYLCCNV viral particle is not infectious to *N*. *benthamiana* by mechanical inoculation therefore the activation of tobacco MAPK could not be monitored at a short time. However, strongly induced NbSIPK at 7-day post argo-inoculation using virus infectious clone still indicates that plant hosts indeed activate the MAPK signaling pathway in response to geminivirus infection (Fig 2A).

### βC1 suppresses the activation of a plant MAPK cascade *in vivo*

Given that geminivirus infection triggers the activation of the plant MAPK defense pathway and that βC1 suppresses MKK2 activity, we hypothesized that βC1 protein might inhibit the MAPK pathway *in vivo*. To test this hypothesis, we treated wild type and βC1-expressing *Arabidopsis* plants with synthetic flg22 peptide, which corresponds to an active epitope of bacterial flagellin. Western blot analysis showed that whereas flg22 treatment elicited a robust MAPK activation in wild type plants, this activation was significantly attenuated in the βC1 transgenic plants (Fig 2B). The phosphoaffinity-based SDS-PAGE approach showed that flg22-activated MPK4 was decreased in βC1-expressing *Arabidopsis* plants, compared with that in wild type plants (S3C Fig). We also treated wild type and βC1-expressing *N*. *benthamiana* plants [35] with synthetic flg22 peptide, and western blot analysis showed that βC1 also inhibits flg22 induced MAPK activation in *N*. *benthamiana* (S3D Fig). These results indicate that βC1 indeed inhibits the flg22-induced MAPK activation *in vivo*.

Two branches of MAPK signaling have been described as key regulators of the transcriptional reprogramming in response to flg22 [24]. One is through MKK4/MKK5-MPK3/MPK6 and can regulate defense gene expression through the transcription factors [14–16], while the other is through MEKK1-MKK1/MKK2-MPK4 and negatively regulates a subset of flg22-induced genes through WRKY33 and the transcriptional repressor ASR3 [21–23]. To study how βC1 impacts the expression of the downstream genes in the MAPK pathway, we selected a number of marker genes and conducted RT-qPCR assays. Notably, the expression of numerous ASR3-suppressed flg22-induced genes, including *AT4G25110* and *AT1G02360* [21] were induced to higher levels in the βC1 transgenic plants compared to the wild type control (Fig 2C). This result suggests that βC1 can release the ASR3-mediated suppression of gene expression. We also selected some other flg22-induced genes that are MPK4-dependent (e.g. *BAP1*, *EXO70B2*) [24, 31], or regulated by both the MPK3/MPK6 and the MPK4 pathways (e.g. *FRK1*, *WRKY22*) [24]. Notably, these genes displayed a significantly lower induction by flg22 in the βC1 transgenic plants relative to the wild type control (Fig 2C). These results suggest that βC1 might interfere with flg22-induced gene reprogramming.

To test how βC1 globally regulates flg22 responsive genes, we performed RNA sequencing (RNA-seq) analysis for wild type and *35S-Myc-βC1* transgenic *Arabidopsis* plants with or without flg22 treatment. Compared with mock treatment, flg22 regulates 1,292 and 1,014 genes in the wild type and *35S-Myc-βC1* transgenic plants, respectively (fold change≥2, P value<0.05) (Fig 2D and S1 and S2 Tables). Strikingly, hierarchical clustering analysis shows that in the *35S-Myc-βC1* transgenic plants, the induction of up-regulated and down-regulated genes in response to flg22 is generally reduced (Fig 2E). Specifically, among the 1,292 flg22-regulated genes, 765 were up-regulated in the wild type. Among these, 43.9% (336/765) were not induced in the flg22 treated *35S-Myc-βC1* line, and 7.1% (54/765) displayed a 1.5-fold higher induction than those in the *35S-Myc-βC1*. On the other hand, among the 527 genes down-regulated in the wild type, 61.7% (325/527) showed no change in the flg22 treated *35S-Myc-βC1* line, and 1.1% (6/527) had 1.5-fold higher reduction than those in the *35S-Myc-βC1* line. These data clearly indicate that βC1 regulates the transcription of both flg22-induced and -repressed genes at the genome-wide scale. Further supporting this notion, Gene Ontology (GO) enrichment analysis of these βC1-regulated genes shows an over-representation of GO categories associated with defense, such as response to chitin, defense response to bacterium, and salicylic acid biosynthetic process (S3E Fig).

In *Arabidopsis*, MAPK activation acts upstream of respiratory burst oxidase homolog D (AtrbohD)-mediated oxidative burst to regulate the deposition of callose [30]. Notably, the flg22-triggered callose deposition was substantially attenuated by βC1 (Fig 2F), consistent with a βC1-mediated attenuation of the MAPK cascade *in vivo*.

### βC1 attenuates Flg22-induced MAPK signaling through inhibition of MKK2

**Fig 3:**
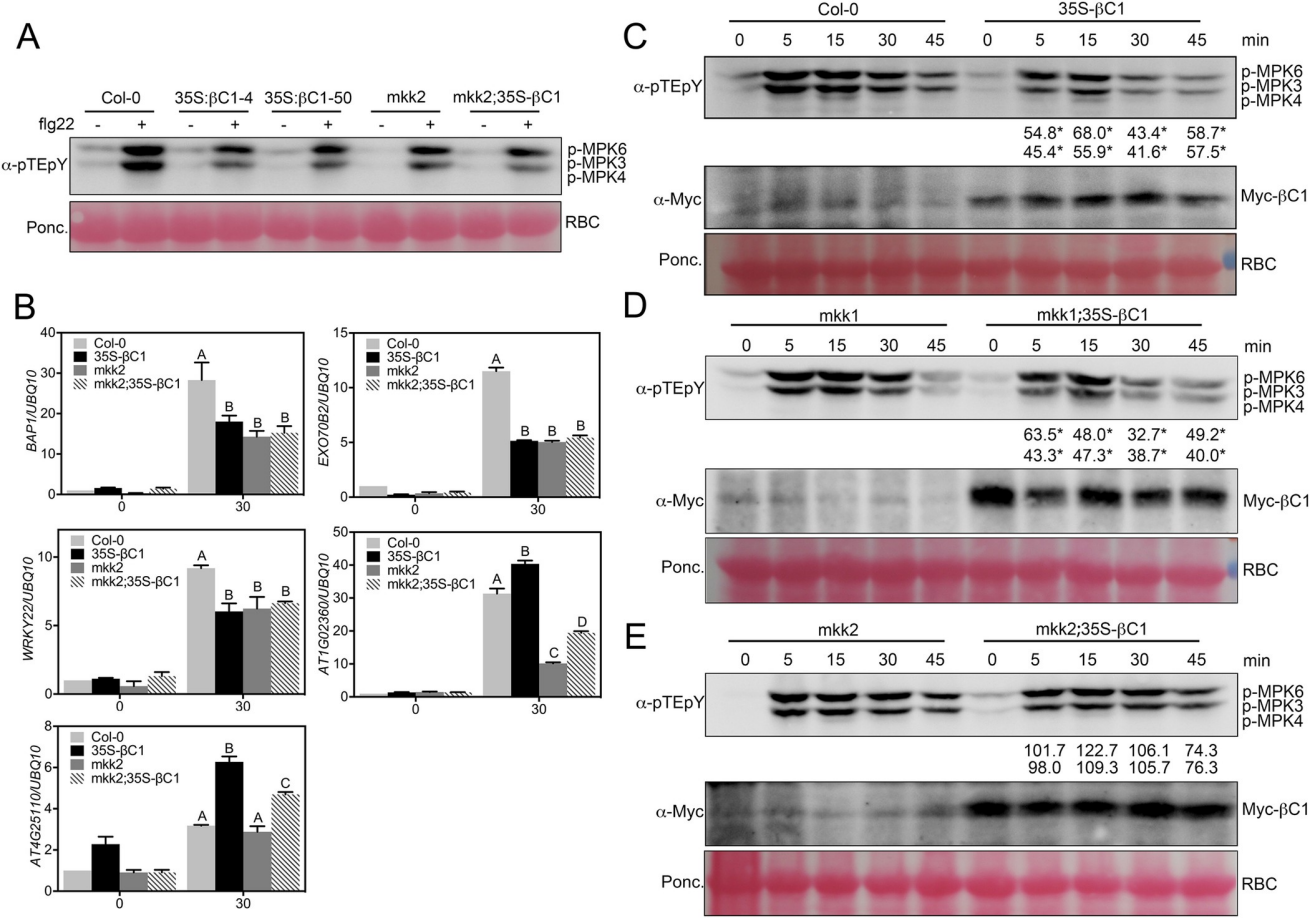
βC1 attenuates flg22 induced MAPK signaling. (**A**) Western blot assays show that differential βC1 attenuation of flg22-induced MAPK activation in Col-0 and *mkk2* background. (**B**) qRT-PCR assays show that βC1 inhibition of flg22-induced gene expression in Col-0 and *mkk2* background. 10-d seedlings were treated with 1μM flg22 for 30 min. Different letters indicate significant differences among samples (p<0.01, t test). (**C**) Western blot assays show that βC1 attenuates flg22-induced MAPK activation *in vivo*. Ten-day-old seedlings were treated with 100 nM flg22 for indicated time windows. MAPK activation was detected by the immunoblot assay with an anti-pTEpY antibody. Myc-βC1 was detected by an anti-Myc antibody and Ponceau S staning of Rubisco (RBC) shows protein loading. (**D**) Western blot assays show that βC1 attenuation of flg22-induced MAPK activation is largely independent on MKK1 protein. (**E**) Western blot assays show that βC1 attenuation of flg22-induced MAPK activation is largely dependent on MKK2 protein. Three biological replicates of these experiments were performed. Numbers indicate the average ratio of phosphorylated MPK6 and MPK3 protein in *35S-Myc-βC1* transgenic plants compared wild type at the same flg22 treatment time period for three biological replicates, asterisks represent statistically significant based on Student’s t test at P < 0.05.

MKK1 and MKK2 function upstream of MPK4 in the MAPK signaling cascade. Previous reports have shown that loss-of-function mutations in either *mkk1* or *mkk2* result in compromised flg22-induced MPK3/MPK6 activation [11, 50]. As previously observed, *mkk2* displayed reduced flg22-induced MPK3/MPK6 activation (Fig 3A), and the transcripts of flg22 responsive genes (*BAP1*, *EXO70B2*, *WRKY22*) accumulated to lower levels upon flg22 treatment (Fig 3B). MKK1 and MKK2 have partially redundant functions in the phosphorylation of MPK4 [11–13]; consequently, MPK4 activation by flg22 remains stable in either *mkk1* or *mkk2* single mutants. Consistent with this, the transcriptional level of MPK4-regulated genes is either similar (*AT4G25110*) or even reduced (*AT1G02360*) in a *mkk2* mutant relative to the wild type control (Fig 3B).

To further investigate how βC1 suppresses the flg22-induced MAPK activation, we transformed the *35S-Myc-βC1* construct into *mkk1* and *mkk2* mutants. Western blot analysis showed that the βC1 protein accumulation was comparable among wild type, *mkk1*, and *mkk2* backgrounds (S3F Fig). In the wild type background, flg22-induced MAPK activation was clearly attenuated in *35S-Myc-βC1* transgenic plants (Fig 3A and 3C). Importantly, the *βC*1-mediated attenuation of MAPK activation was clearly observed in *mkk1*, but not in the *mkk2* background (Fig 3D and 3E). Furthermore, no significant impact on transcript accumulation of flg22-induced genes by βC1 was detected in *mkk2* plants (Fig 3B), also the transcription level of the MPK3, MPK4 and MPK6 was not affected by βC1 (S4 Fig). Thus, these results, together with the specific βC1-MKK2 interaction *in vitro* and *in vivo*, indicated that βC1 inhibits flg22-induced MAPK activation specifically through MKK2, but not through MKK1.

### MKK2 is involved in defense against geminiviruses in both *Arabidopsis* and tobacco

If MKK2 is a key component of the plant defense against geminiviruses and the βC1-mediated suppression of the MKK2 function represents a counter-defense mechanism, one would expect a higher sensitivity of the *mkk2* mutant to viral infection. We initially tested this hypothesis in *Arabidopsis*, and noticed that *Arabidopsis* infected with TYLCCNV, with or without TYLCCNB, did not display obvious symptoms (S5A Fig). In spite of this, the infectious rate of TYLCCNV with betasatellite was significantly higher in *mkk2* plants than that in the wild type (S5B Fig).

**Fig 4:**
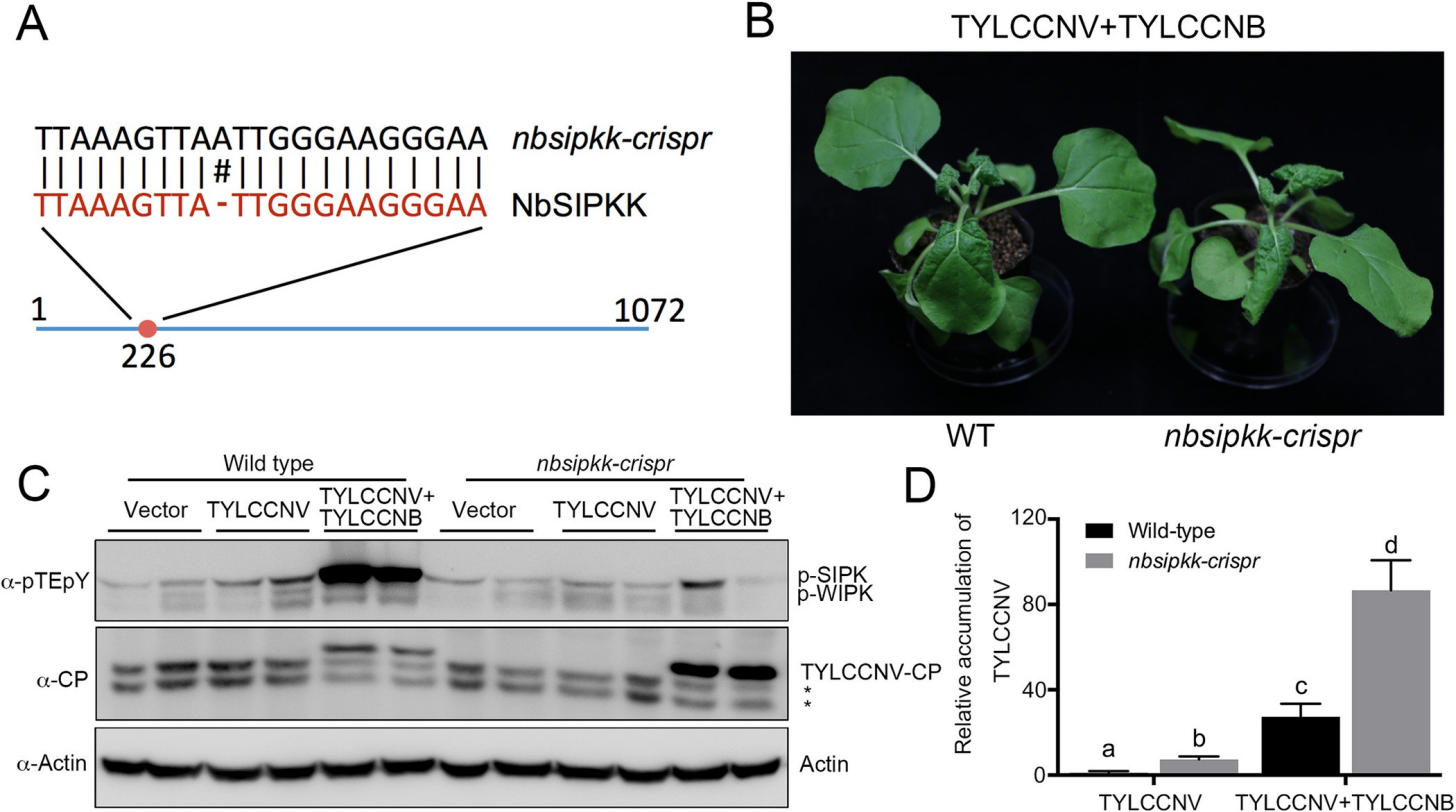
MKK2 participates in basal defense against virus. (**A**) Location of single guide RNA target in *NbSIPKK* locus. An additional adenine was inserted into the exon of *NbSIPKK*, leading to the frame shift in the coding sequence. (**B**) *nbsipkk-crispr* plants displayed severe curl leaves with virus infection. Eight-leaf-period wild type and *nbsipkk-crispr N*. *benthamiana* leaves were infiltrated with *A*. *tumefaciens* harboring either TYLCCNV+TYLCCNB infectious clone, or empty vector. Plant phenotype was monitored 7 days after inoculation. (**C**) Western blot analysis of MAPK activation and virus titers of systemic leaves in wild-type or *nbsipkk-crispr* plants. Endogenous phosphorylated NbSIPK was monitored by immunoblot with an anti-pTEpY antibody. Virus amount was detected by the immunoblot assay with a TYLCCNV-CP antibody. (**D**) Relative accumulation levels of TYLCCNV in agroinfiltrated wild type and *nbsipkk-crispr* plants. Viral accumulation was determined by qPCR at 7 dpi. The values represent viral DNA accumulation relative to levels in TYLCCNV infected wild type plants. The data are shown as means and standard error of the mean (SEM) of three biological replicates. Different letters indicate significant differences among samples (p<0.05, Student’s t test).

**Fig 5:**
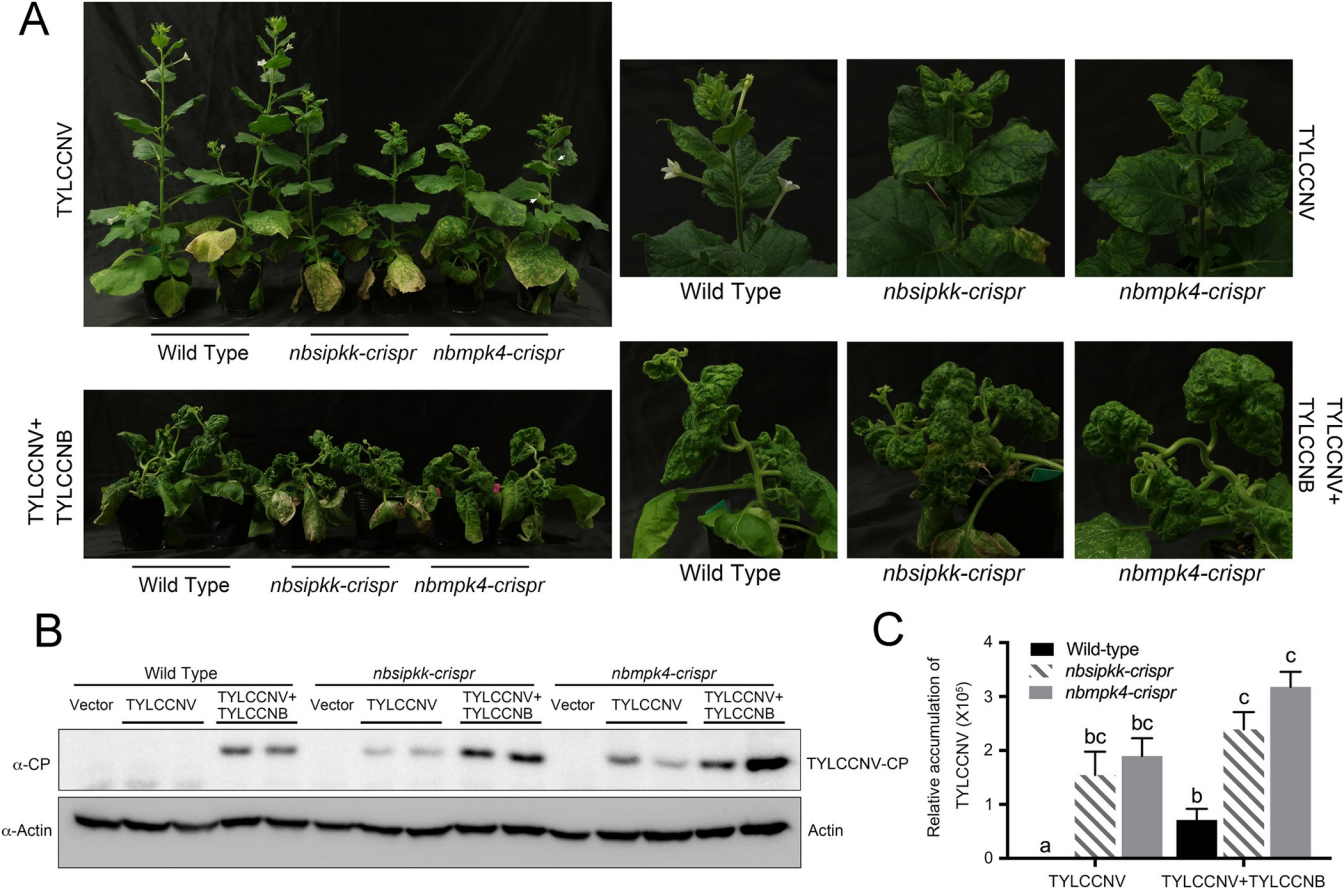
MKK2 and MPK4 participate in basal defense against virus. (**A**) *nbsipkk-crispr* plants and *nbmpk4-crispr* plants displayed severe growth defect with TYLCCNV and TYLCCNV/TYLCCNB infection. Eight-leaf-period wild-type, *nbsipkk*-*crispr* and *nbmpk4-crispr N*. *benthamiana* leaves were infiltrated with *A*. *tumefaciens* harboring TYLCCNV or TYLCCNV/TYLCCNB infectious clone. Plant phenotype was monitored 30 days after inoculation. (**B**) Western blot analysis of virus titers in wild type, *nbsipkk-crispr* or *nbmpk4-crispr* plants. Virus amount was detected by the immunoblot assay with a TYLCCNV-CP antibody. (**C**) Relative accumulation levels of TYLCCNV in agroinfiltrated wild type, *nbsipkk-crispr* or *nbmpk4-crispr* plants. Viral accumulation was determined by qPCR at 30 dpi. The values represent viral DNA accumulation relative to levels in TYLCCNV infected wild type plants. The data are shown as means and SEM of three biological replicates. Different letters indicate significant differences among samples (p<0.05, Student’s t test).

To further test this hypothesis, we used the CRISPR/Cas9 system to knockout *NbSIPKK* in *N*. *benthamiana*. Sequencing analysis revealed that the mutagenesis introduced a nucleotide into the *NbSIPKK* locus and caused a frame shift (Fig 4A). Thus, our *nbsipkk-crispr* line is a null-allele. We then inoculated these mutant plants with the virus or empty vector: seven days after inoculation with TYLCCNV+TYLCCNB, *nbsipkk-crispr* plants showed more strongly curled leaves compared to the wild type plants (Fig 4B), and significantly reduced NbSIPK activation and higher virus accumulation (Fig 4C). Increased viral load was also observed in systemic infected *nbsipkk-crispr* leaves by western blot against the viral coat protein and qPCR assay at 30 days post inoculation (Fig 5B and 5C). Notably, 30 days after inoculation with TYLCCNV only, *nbsipkk-crispr* plants showed stunted growth, curled leaves, and arrested flower development (Fig 5A), reminiscent of what could be observed in TYLCCNV+TYLCCNB-infected wild type plants. Virus accumulation was higher in TYLCCNV-infected *nbsipkk-crispr* than in wild type (Fig 5B and 5C). Vector-inoculated *nbsipkk-crispr* or wild type plants did not exhibit growth abnormalities. These results indicate that MKK2 is indeed involved in defense against geminiviruses in both *A*. *thaliana* and *N*. *benthamiana*.

### βC1 interacts with MPK4 in the nucleus and inhibits MPK4 kinase activity

**Fig 6:**
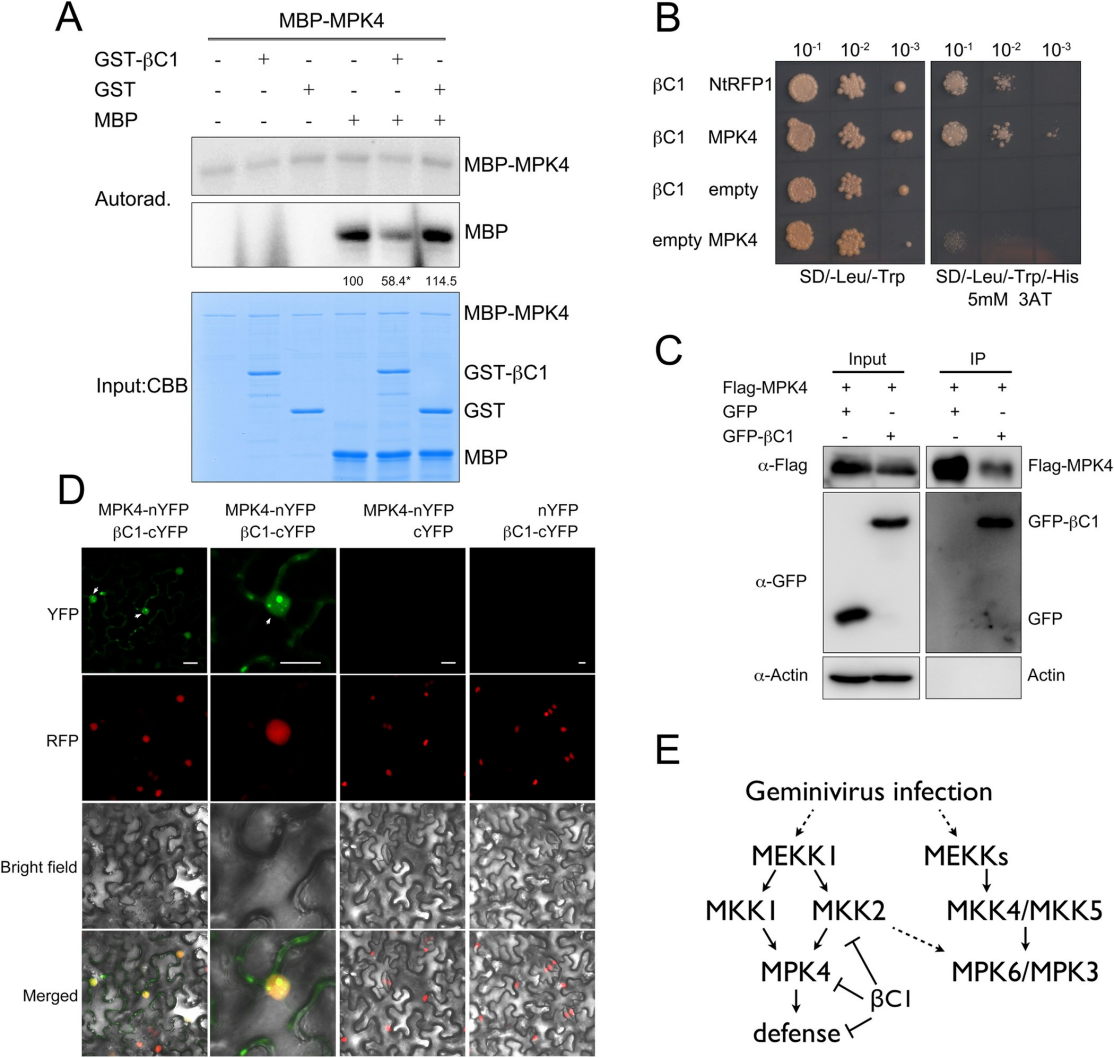
βC1 physically interacts with MPK4. (**A**) *In vitro* immunocomplex kinase assays show that βC1 suppresses MPK4 activity. Different purified kinases, myelin basic protein (MBP) substrates, and [γ-32P] ATP were incubated with either GST-βC1 or GST protein. Proteins were resolved in SDS-PAGE and analyzed by autoradiography. Protein loading is shown by Commassie Brilliant Blue staining (CBB). Numbers indicate relative amount of phosphorylated MBP protein of three biological replicates. Asterisk indicates significant differences (p<0.05, Student’s t test). (**B**) Y2H assay shows that βC1 interacts with MPK4. Yeast transformant harboring different combinations of AD-βC1 and BD-MPK4 were spotted with 10-fold serial dilutions on SD/-Leu/-Trp medium and SD/-Leu/-Trp/-His containing 5mM 3-AT. βC1 and NtRFP1 serve as positive control. (**C**) Co-IP assay confirmed the *in planta* interaction between βC1 and MPK4. Samples before (Input) and after (IP) immunopurification were analyzed by immunoblot using anti-GFP and anti-Flag antibodies, actin serves as a control. (**D**) BiFC assay validates the interaction between MPK4 and βC1. RFP-H2B transgenic *N*. *benthamiana* leaves were infiltrated with *A*. *tumefaciens* harboring combinations of indicated constructs. Columns from left to right represent YFP fluorescence, RFP fluorescence, bright field and YFP/RFP/bright field overlay. Bars represent 20μm. (**E**) The proposed model of βC1 effect on MAPK cascade in virus defense. Geminivirus infection induces activation of MPK6/MPK3 and MPK4, βC1 blocks MAPK cascade regulated defense by inhibiting MKK2 and MPK4.

MPK4 locates downstream of MKK2 and plays a key role in regulating host defense [5, 10, 12–13]. To further investigate whether βC1 alters MPK4 kinase activity, we conducted the kinase reconstitution assay with a sensitive MAPK substrate, myelin basic protein (MBP), which could be phosphorylated by purified MPK4 (Fig 6A). Interestingly, GST-βC1, but not GST alone, significantly decreased the phosphorylation signal.

The clear βC1-mediated inhibition of MPK4 kinase activity prompted us to hypothesize that βC1 might associate with MPK4 through protein-protein interaction. In order to test this idea, we carried out Y2H, Co-IP, and LCI assays with βC1 and MPK4. As shown in Fig 6B and 6C and S6A Fig, all of the assays demonstrated that MPK4 and βC1 proteins indeed interact with each other *in vitro* and *in planta* (Fig 6B and 6C and S6A Fig). Furthermore, BiFC assays showed that the interaction occurs mainly at nuclear speckles (Fig 6D).

MPK4 is closely related to ERK2 in mammals, which is known to self-dimerize and relocate to the nucleus upon phosphorylation [51]. Similarly, we observed that MPK4 localizes to the cytoplasm and nucleus, while a MPK4 homodimer is found exclusively in the nucleus (S6B and S6C Fig). The similar nuclear localization pattern of the βC1/MPK4 interaction and the MPK4 homodimer suggested that βC1 might target the phosphorylated form of MPK4. We also noticed that the MPK4-regulated genes were repressed in the *βC1* transgenic plants. For example, the transcription level of flg22-induced ASR3-suppressed genes (*AT1G02360*, *AT4G25110*) [21] was increased in *mkk2; 35S-βC1* compared to *mkk2* (Fig 3B), suggesting that βC1 could target MPK4 directly *in vivo* upon flg22 treatment.

### MPK4 is involved in defense against geminiviruses in *N*. *benthamiana*

To further investigate the functional relevance of the βC1-mediated inhibition of MPK4, we used the CRISPR/Cas9 system to knockout tobacco *NbMPK4* (Niben101Scf07241g00013.1), which is homolog to *AtMPK4* and can be phosphorylated by SIPKK *in vivo* [52]. We identified the direct interaction between NbMPK4 and βC1 by Co-IP assay (S7A Fig). Sequencing analysis showed that the *nbmpk4-crispr* line had 65 nucleotides missing in the fourth exon of *NbMPK4* (S7B and S7C Fig). Unlike *mpk4* mutant *Arabidopsis* that shows strong dwarf phenotype, *nbmpk4-crispr* plants did not show any developmental defects, probably because of the existence of redundant genes [53]. However, like *nbsipkk-crispr* plants, *nbmpk4-crispr* plants displayed stunted growth, curled leaves and arrested flower development when infected by TYLCCNV (Fig 5A). In addition, we found curly anthocauli in TYLCCNV-infected *nbmpk4-crispr* plants. The phenotypical similarity between TYLCCNV-infected *nbmpk4-crispr* and *nbsipkk-crispr* plants suggests that these two genes participate in the same defense pathway. Moreover, virus accumulation in the *nbmpk4-crispr* systemic leaves, infected with either TYLCCNV or TYLCCNV+TYLCCNB, is higher than that in wild type plants (Fig 5B and 5C). Compared with TYLCCNV infected wild type plants, a significant higher amount of viral DNA was accumulated in TYLCCNV+TYLCCNB infected plant. However, the levels of viral DNA were comparable between TYLCCNV and TYLCCNV+TYLCCNB infected *nbmpk4-crispr* and *nbsipkk-crispr* plants, indicating that βC1 might promote virulence more in wild-type plants than in *nbmpk4-crispr* and *nbsipkk-crispr* plants (Fig 5C). Altogether, our virus infection assays support that the MKK2-MPK4 cascade is one line of defense against geminiviruses in *N*. *benthamiana* (Fig 6E).

## Discussion

Plants have evolved multiple strategies and tactics to combat invading pathogenic viruses. Here, we report a new battlefield between host defense and geminivirus counter-defense. We propose that plants activate the MAPK pathway to defend themselves against geminivirus infection; on the other end, successful viruses have evolved to suppress this response and thus promote viral infection, as we have shown for βC1 (Fig 6E). Several pieces of evidence support this model: 1) virus infection induces the activation of a tobacco MAPK kinase (SIPK) in a natural host (Fig 2A); 2) Lack of SIPKK or MPK4 can complement the lack of βC1. TYLCCNV-infected *nbsipkk-crispr* and *nbmpk4-crispr* knockout tobacco plants show the typical symptoms of TYLCCNV+TYLCCNB-infected plants (Fig 5A); 3) higher virus accumulation is detected in *nbsipkk-crispr* and *nbmpk4-crispr* tobacco (Fig 5B and 5C); 4) a higher virus infectious rate is observed in TYLCCNV+TYLCCNB-inoculated *mkk2* mutant than in wilt type *Arabidopsis* (S5B Fig); 5) βC1 interacts with MKK2 and MPK4 *in planta* (Figs 1B–1E, 6B and 6D and S6A and S7A Figs); 6) βC1 blocks MKK2 and MPK4 kinase activity (Figs 1F and 6A); 7) βC1 inhibits downstream responses of the PAMP-induced MPK6/MPK3/MPK4 activation *in vivo*, namely the expression of downstream MAPK-regulated genes and callose deposition (Fig 2B–2F). To the best of our knowledge, this report represents the first evidence of a MAPK cascade involved in defense against geminiviruses; moreover, it describes a novel mechanism of an inhibition of this defense pathway by a viral protein through the simultaneous direct targeting of multiple components of this cascade.

The MAPK cascade serves as an important line of defense activated upon perception of potential pathogens in both animals and plants. Early studies have demonstrated that MAPK cascade is associated with plant virus infection. For instance, MKK4, MKK5, and MPK6 transcripts are elevated in cabbage leaf curl virus (CaLCuV)-infected *Arabidopsis* [54]. In tobacco, WIPK/SIPK is activated upon the infection of tobacco mosaic virus (TMV) [32, 49]. In line with these reports, we detected a potent activation of NbSIPK in TYLCCNV+TYLCCNB-infected *N*. *benthamiana* plants. These results imply that MAPK activation might be a conserved response to virus infection. Although βC1 could inhibit MAPK activation in *Arabidopsis* and *N*. *benthamiana*. Higher MAPK activation could be detected in TYLCCNV+TYLCCNB-infected *N*. *benthamiana* plants than that in TYLCCNV infected plants. We hypothesized it is the result from the increased viral reproduction in the presence of βC1. Notwithstanding, the mechanism by which viruses could elicit MAPK activation during the infection awaits further elucidation. Since viruses are obligate intracellular pathogens, it was traditionally assumed that PTI, which is considered to be initiated by perception of extracellular elicitors by PRRs, would not play a role in antiviral defense. However, recent years’ mounting evidence points at a contribution of receptor-like proteins and other elements required for PTI signaling in the defense against plant viruses [55, 56]. Loss-of-function mutations in the PRR co-receptors BAK1 and BAK1-LIKE1 (BKK1) result in enhanced susceptibility to plum pox virus (PPV) [55]. In addition, pre-treatment with double-stranded RNA induces plant resistance against oilseed rape mosaic virus (ORMV) [56]. These observations strongly support a potential role of PTI in plant anti-viral defense. In the future, it will be essential to identify the viral component(s) responsible for eliciting MAPK activation, and the host receptor(s) responsible for the recognition, in order to further shed light on this front of the interaction between plant and virus.

MAPK cascades are involved in both PTI and ETI, and play a pivotal role in regulating phytoalexin biosynthesis and defense gene expression [4–5, 10]. The battle between plant MAPKs and pathogen effectors has been mainly reported for pathogenic bacteria [28–31]. Here, we demonstrate that this strategy is also used by plant viruses. Most bacterial effectors perturb the MAPK cascade through specific enzymatic activity. The *P*. *syringae* effector HopAI1 acts as a novel phosphatase that cleaves the C-OP bound to the phosphothreonine of the TXY motif in the activation loop of MAPKs [30]; HopF2 possesses ADP-ribosyltransferase activity to modify and block MKK5 [29]. Unlike pathogenic bacteria, which deliver approximately 20–30 effectors into host cells, plant viruses have a compact genome organization, with the concomitant coding restrictions, and therefore encode limited but multi-functional proteins. Most geminiviral proteins do not possess demonstrated enzymatic activity toward host proteins. Therefore, we hypothesize that the inhibitory activity of βC1 on MKK2 and MPK4 most likely occurs through direct binding to the protein, which would block the kinase domain or the activation loop. Intriguingly, we observed that a subset of flg22-induced genes was down-regulated in *35S-βC1* plants in the absence of flg22 treatment (Fig 3F), suggesting that the basal level of defense is already repressed by βC1.

The betasatellite-encoded βC1 protein has been reported to interfere with host hormone signaling, which leads to a promotion of virus vector performance [36, 38, 43]. TYLCCNB-encoded βC1 protein can reduce the transcriptional level of several JA-responsive genes through targeting AS1 and promote its repressive activity in JA pathway [38]. Also βC1 interacts with a basic helix-loop-helix transcription factor MYC2 to regulate a subset of JA-mediated gene expression [43]. Accordingly, repressed JA responses and elevated SA-responsive gene expression were found in TYLCCNV/TYLCCNB infected plants [57]. Similarly, *mpk4* knockout *Arabidopsis* is insensitive to JA treatment, and exhibits constitutive activation of SA-dependent defenses [52, 58]. Therefore, future work will be required to determine whether altered hormone signaling caused by βC1 results from its inhibition of MAPK cascades.

## Materials and methods

### DNA constructions

Coding sequences of genes were subcloned into pENTR-D-TOPO vector (Thermo Fisher Scientific). Plant constructs (pBA-Flag-Myc4-DC, pER10-YFP-DC) [44] and *Escherichia coli* expression recombinant protein constructs (pMAL-MBP-DC, pGEX4T-3-GST-DC) were made using the Gateway system (Invitrogen, Carlsbad, CA, US). For details, primers and constructs used in this study are listed in S3 Table. Site-directed mutagenesis was performed as described. Plant constructs were transformed into *Agrobacterium tumefaciens* strain C58C1 (for BIFC assay) and GV3101 (for LCI assay).

### Yeast two-hybrid assay

Full-length *TYLCCNB βC1* was PCR amplified and cloned into yeast vector pGADT7 to generate the DNA activation domain (AD)-containing vector pGADT7-βC1. The full-length *MKK1*, *MKK2*, *MPK4* were PCR amplified and cloned into binding domain (BD) containing vector pGBKT7. Plasmids pGADT7-βC1 and pGBKT7-candidates were co-transformed into *Saccharomyces cerevisiae* Gold strain according to Yeastmaker Yeast Transformation System 2 (Clontech). Transformants were grown on synthetic medium -Leu/-Trp at 30°C for 72 h and then transferred to the selective medium -Leu/-His/-Trp containing 5 mM 3-aminotriazole (3-AT) to identify binding activity. Interaction between βC1 and NtRFP1 serves as the positive control [46].

### Plant materials and growth conditions

*A*. *thaliana* mutant *mkk2* (SALK_200520) was provided by Dr. Ping He (Texas A&M University, TX, US), *mkk1* (SALK_027645) was provided by Dr. Yueling Zhang (University of British Columbia, BC, CA). 35S: βC1 transgenic *N*. *benthamiana* was generated before [35]. *nbsipkk-crispr* and *nbmpk4-crispr* transgenic *N*. *benthamiana* was generated by transformation with binary vector BGK01-NbSIPKK and BGK01-NbMPK4 (Biogle, Hangzhou, CHN). Single guide RNA targeting open reading frame of NbSIPKK and NbMPK4 was designed using CRISPR-P tool (http://cbi.hzau.edu.cn/crispr/). T2 homozygous transgenic plants were used and confirmed positive before virus inoculation assay.

*A*. *thaliana* plants were grown in the growth room at 22°C, 50% humidity with a 12h/12h photoperiod. Seedlings were germinated on full-strength Murashige and Skoog medium containing 1% sucrose and 0.8% agar, grown under the same conditions for 10d, transferred to a six-well tissue culture plate with 2 mL water overnight, and then treated with flg22 peptide for indicated time for MAPK and qRT-PCR assays. *N*. *benthamiana* plants were placed in the 25°C and 60% humidity with a 16h/8h photoperiod.

### MAPK assay

Ten-day-old *Arabidopsis* or eight-day-old *N*. *benthamiana* seedlings were transferred from full-strength Murashige and Skoog medium to water for overnight, and then treated with 100nm flg22 for indicated time points. Seedlings were ground in extraction buffer (100 mM HEPES, pH 7.5, 10 mM NaF, 5 mM EDTA, 5mM EGTA 10mM Na_3_VO_4_ 10% glycerol, 10 mM DTT and 1mM PMSF. And protein samples were separated in SDS-PAGE gel to detect phosphorylated MAPK by immunoblotting with anti-pTEpY antibody [21] (Cell Signaling, MA, USA).

### Virus inoculation

Eight-leaf-period *N*. *benthamiana* plants and three-week-old Col-0 wild-type, *mkk2* mutant *Arabidopsis* plants were used for virus infection. Equal volumes of individual *A*. *tumefaciens* cultures at an OD600 = 1.0 were mixed prior to infiltration. Viral infectious clones of TYLCCNV and TYLCCNB [35] were described previously. Systemic leaves (the third and fourth leaves from top) were collected for further analysis.

### Luciferase complementation imaging (LCI) assay

4-week-old *N*. *benthamiana* leaves were infiltrated with combinations of *A*. *tumefaciens* harboring an equal volume of the derivative constructs of pER10-CLuc and pCambia-NLuc. The LCI assays were then performed as described previously [44]. Catalytic activity of luciferase in each combination was recorded through CCD camera. CLuc fused cucumber mosaic virus-encoded 2b protein and NLuc fused *Arabidopsis* Argonaute1 (Ago1) were used as the positive control in this assay [47].

### BiFC assay

4-week-old *N*. *benthamiana* leaves were co-infiltrated with various BiFC constructs as shown in the figures. BiFC assays were performed as described previously [46].

### Co-IP assay

4-week-old *N*. *benthamiana* leaves were co-infiltrated with indicated constructs as shown in the figures. Total protein was extracted with the IP buffer containing 40 mM Tris·HCl, pH 7.5, 150 mM NaCl, 5 mM MgCl_2_, 2 mM EDTA, 5 mM DTT, 0.5% Triton X-100, 5% glycerol, 2mM PMSF and EDTA-free protease inhibitor mixture (Roche, Basel, CH). Soluble proteins were cleared by centrifugation and then immunoprecipiated with Anti-FLAG M2 Magnetic Beads (Sigma–Aldrich, MO, USA). Co-IP assay was then performed as described [46].

### Quantitative RT-PCR

Total RNA from *Arabidopsis* seedlings was isolated using TRIzol (Invitrogen, CA, USA). 1 μg RNA sample was reverse transcribed with the ReverTra Ace qPCR RT MasterMix (TOYOBO, Osaka, JPN). Real-time PCR was conducted using the Universal SYBR Green Master (Roche, Basel, CH) with reported gene-specific primers [14, 21–22].

### Immuno-precipitation for βC1 complex isolation

4-week-old *N*. *benthamiana* leaves were infiltrated with combinations of *A*. *tumefaciens* harboring Flag-4Myc-βC1 under the control of cauliflower mosaic virus 35S promoter. Infiltrated leaves were collected and extracted using four volumes of extraction buffer [40 mM Tris-HCl, pH 7.5, 150 mM NaCl, 5 mM MgCl_2_, 2 mM EDTA, 5 mM DTT, 0.1% Triton X-100, 2% glycerol, 2mM PMSF and EDTA-free protease inhibitor mixture (Roche, Basel, CH)]. Then βC1 complex was isolated and analyzed as indicated [44].

### RNA sequencing analysis

Total RNA of wild type and 35S-βC1 with or without flg22 treatment samples (three independent biological repeats) were extracted using Trizol reagent (Invitrogen, CA, USA) following the manufacturer's procedure. The cDNA library was created in accordance with the protocol for the mRNA-Seq sample preparation kit (Illumina, San Diego, USA) and the average insert size for the paired-end libraries was 300 bp (±50 bp). The paired-end sequencing was then performed on an Illumina Hiseq 4000 at the (LC-bio, Hangzhou, CHN) following the vendor’s recommended protocol. About 50 million valid reads were obtained for each sample. These clean reads were aligned to the *Arabidopsis* reference genome (TAIR10) using Tophat package. StringTie was used to assemble the mapped reads, estimate the expression levels of all transcripts and calculate the number of fragments per kilobase of exon model per million mapped reads (FPKM), in order to select differentially expressed mRNAs (log2 (fold change) >1 or log2 (fold change) <-1, with statistical significance (p value < 0.05)). Clustering heat map for flg22 regulated genes in wild type was generated using the Mev4 (http://www.tm4.org/mev.html).

## Supporting information

S1 Table List of flg22-regulated genes in wild type Arabidopsis. (XLSX)

S2 Table List of flg22-regulated genes in 35S-Myc-βC1. (XLSX)

S3 Table List of primers used in this study. (XLSX)

S1 Fig Identification of MKK2 as a new bona fide target of TYLCCNB βC1. (**A**) Peptides uniquely matched to NbSIPKK were recovered from proteomic analysis of βC1 complex. (**B**) Phylogenetic tree of tobacco and *Arabidopsis* mitogen-activated protein kinase kinases (MAPKKs) was created using Jotun Hein method based on the entire amino acid sequence of each MAPKK. *A*. *thaliana At*MKK1 (AT4G26070), AtMKK2 (AT4G29810), *Nicotiana tabaccum* NtMEK1/NQK1 (AB055514), NtMEK2 (AB264547), NtSIPKK (NM_001326032), *Nicotiana attenuata* NaMKK1 (NW_017670940), NaMEK2 (NC_031991). (**C**) The interaction between βC1 and NbSIPKK was confirmed by Co-IP assay. *N*. *benthamiana* leaves were infiltrated with *A*. *tumefaciens* cells harboring 3Flag-NbSIPKK with GFP-βC1 or GFP for Co-IP assay. Samples before (Input) and after (IP) immunopurification were analyzed by immunoblot using anti-GFP and anti-Flag antibody. (**D**) The protein level of MKK1-nYFP, MKK2-nYFP and βC1-cYFP in the BiFC assay were shown by immunoblotting using anti-HA antibody. Combinations of agro-infiltrated constructs were indicated. Actin serves as a control.
(JPG)

S2 Fig βC1 protein interacts with the kinase domain of MKK2. (**A**) Diagram of MKK2 truncated or deletion variants. (**B**) BiFC visualization of interaction between MKK2 mutants and βC1 in 35S-*RFP-H2B* transgenic *N*. *benthamiana* leaves. Combinations of the infiltrated constructs were indicated. Columns from left to right represent fluorescence of YFP, and RFP fluorescence, bright field and YFP/RFP/bright field overlay, respectively. Bars represent 50 μm. (**C**) The protein level of MKK2-NTP-nYFP, MKK2-CTP-nYFP, MKK2-KD-nYFP and βC1-cYFP in the BiFC assay were shown by immunoblotting using anti-HA antibody. Combinations of agro-infiltrated constructs were indicated. Actin serves as a control.
(JPG)

S3 Fig βC1 inhibits the MAPK cascade that is activated by virus infection. (**A**) Viral accumulation was determined by qPCR. The values represent viral DNA accumulation relative to level in TYLCCNV infected plants. The data are shown as means and SEM of three biological replicates. Asterisk indicates significant difference (p<0.05, Student’s t test). (**B**) The mRNA level of CP and βC1 were shown by RT-PCR, Actin serves as a control. (**C**) Flg22-induced MPK4 activation in Wild type and *35S-βC1 Arabidopsis*. 10-day seedlings were treated with 100 nM flg22 for 15 min and subjected to immunoblot assays with an anti-pTEpY, anti-MPK4 or anti-Myc antibody. In phosphoaffinity-based SDS-PAGE, total protein were separated in a 10% SDS-PAGE gel supplemented with 50 mM phos-tag (Wako chemicals USA, Inc.), and MPK4 and p-MPK4 protein were detected with anti-MPK4 antibody. Three biological replicates were performed. Numbers indicate the average amount of phosphorylated MPK4 protein, and the values were normalized to flg22 treated wild-type samples. Asterisk indicates significant differences (p<0.05, Student’s t test). (**D**) Flg22-induced MAPK activation in Wild type and *35S-βC1* transgenic *N*. *benthamiana*. 8-day seedlings were treated with 100 nM flg22 for indicated time period and subjected to immunoblot assays with an anti-pTEpY, anti-βC1 or anti-Actin antibody. Accumulated phosphorylated NbSIPK level for three biological replicates was calculated and is shown below. (**E**) GO enrichmentanalysis of genes that were either significant up/down regulated in wild-type but not in *35S-Myc-βC1* or significant up/down regulated in *35S-Myc-βC1* but had a 1.5 times lower fold change than wild-type. (**F**) Amount of Myc-βC1 protein in wild type, *mkk1* and *mkk2* background. Total protein of 10-day T2 homozygous transgenic seedlings was subjected to immunoblot assays with an anti-Actin or anti-Myc antibody. Ponceau S staining of Rubisco (RBC) shows protein loading.
(TIF)

S4 Fig RT-qPCR analysis of *MPK3, MPK4* and *MPK6*. RT-qPCR analysis of MPK3, MPK4 and MPK6 in Col-0 and 35S-Myc-βC1 with or without flg22 treatment for 30 min. The letters indicate significant differences with a Student’s t test (P < 0.05).
(JPG)

S5 Fig MKK2 participates in basal defense against virus. (**A**) TYLCCNV infected *Arabidopsis* did not exhibit developmental defect. Eight-leaf-period *Arabdopsis* seedlings were inoculated with *A*. *tumefaciens* harboring TYLCCNV/TYLCCNB, TYLCCNV infectious clone or empty vector, respectively. Phenotype was monitored 8 days post infiltration. Bars represent 2cm. (**B**) Virus CP gene of 20 TYLCCNV+TYLCCNB inoculated wild type or *mkk2* mutant *Arabidopsis* plants was analyzed by PCR. Actin serves as a loading control.
(JPG)

S6 Fig In vivo interaction of βC1/MPK4, MPK4/MPK4 and cellular distribution of MPK4. (**A**) LCI assay shows that βC1 interacts with MPK4 in planta. Different Combinations of NLuc and CLuc derivative constructs were co-infiltrated into *N*. *benthamiana* leaves for LCI assay. Infiltrated positions on the leaf were shown in the left panel. Fluorescence signal represents protein-protein interaction. Bar represents 5cm. (**B**) *A*. *tumefaciens* harboring GFP-MPK4 was infiltrated into *N*. *benthamiana* GFP fluorescence was analyzed using confocal microscopy. Bars represent 50 μm. (**C**) *A*. *tumefaciens* harboring combinations of indicated constructs were infiltrated into RFP-H2B transgenic *N*. *benthamiana* leaves. YFP or RFP fluorescence was analyzed using confocal microscopy. Columns from left to right represent YFP fluorescence, RFP fluorescence, bright field and YFP/RFP/bright field overlay. Bars represent 50 μm.
(JPG)

S7 Fig Nucleotide deletion of *nbmpk4-crispr N. benthamiana*. (**A**) Confirm the interaction between βC1 and NbMPK4 by Co-IP assay. *N*. *benthamiana* leaves were infiltrated with *A*. *tumefaciens* cells harboring 3Flag-NbMPK4 with GFP-βC1 or GFP for Co-IP assay. Samples were analyzed by immunoblot using anti-GFP and anti-Flag antibody (**B**) Location of single guide RNA target in *NbMPK4* locus. 65 nucleotides were deleted in the exon of *NbMPK4*. (**C**) PCR analysis of a 284 nt long sequence which includes single guide RNA target region in *NbMPK4* locus.
(JPG)
