# Supplementary figures and images for: βC1 protein encoded in geminivirus satellite concertedly targets MKK2 and MPK4 to counter host defense
Source: PLoS Pathog. 2019 Apr 18;15(4):e1007728. doi: 10.1371/journal.ppat.1007728 (PMC6499421; doi:10.1371/journal.ppat.1007728)

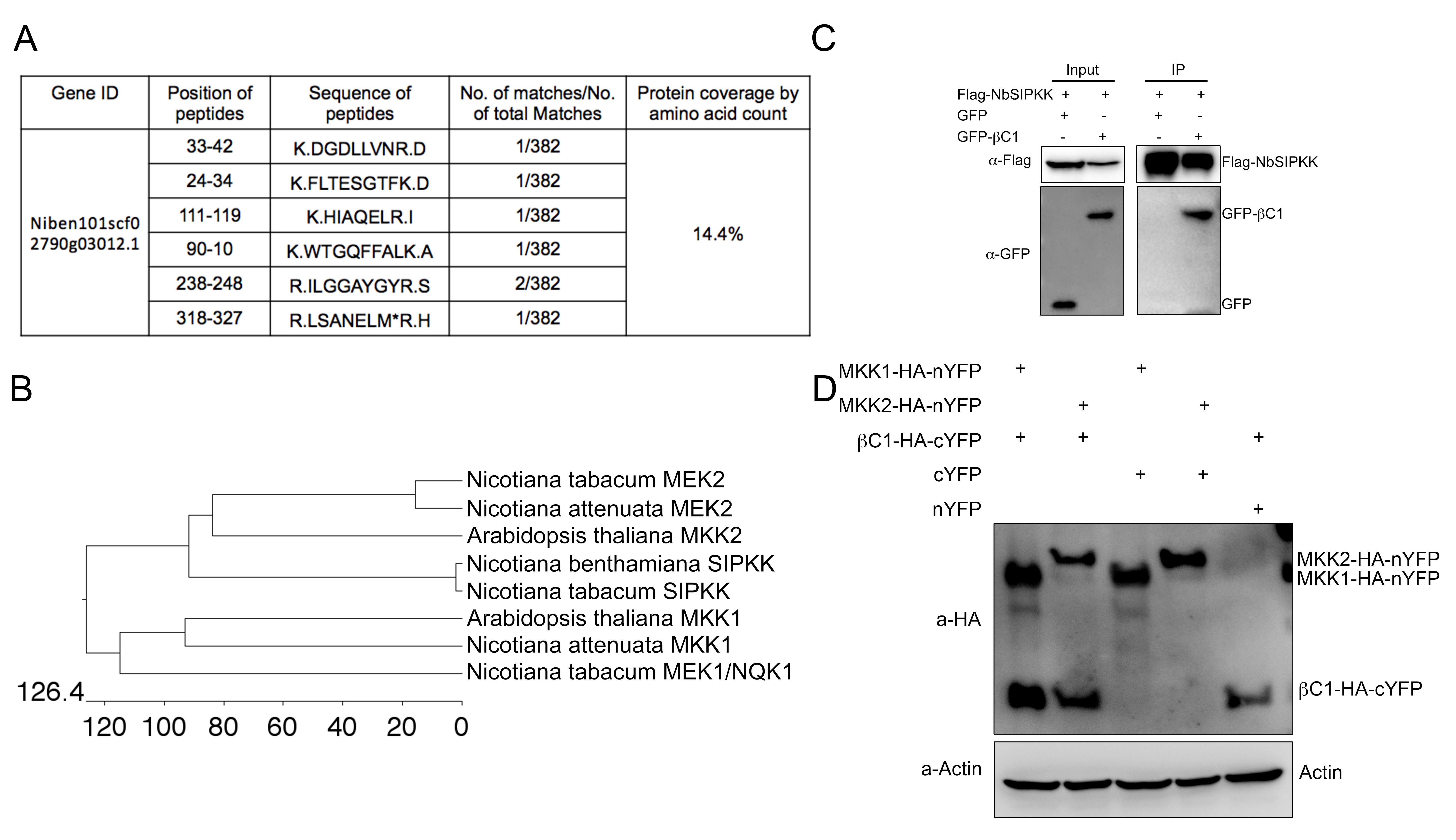

Supplement: S1 Fig — (A) Peptides uniquely matched to NbSIPKK were recovered from proteomic analysis of βC1 complex. (B) Phylogenetic tree of tobacco and Arabidopsis mitogen-activated protein kinase kinases (MAPKKs) was created using Jotun Hein method based on the entire amino acid sequence of each MAPKK. A. thaliana AtMKK1 (AT4G26070), AtMKK2 (AT4G29810), Nicotiana tabaccum NtMEK1/NQK1 (AB055514), NtMEK2 (AB264547), NtSIPKK (NM_001326032), Nicotiana attenuata NaMKK1 (NW_017670940), NaMEK2 (NC_031991). (C) The interaction between βC1 and NbSIPKK was confirmed by Co-IP assay. N. benthamiana leaves were infiltrated with A. tumefaciens cells harboring 3Flag-NbSIPKK with GFP-βC1 or GFP for Co-IP assay. Samples before (Input) and after (IP) immunopurification were analyzed by immunoblot using anti-GFP and anti-Flag antibody. (D) The protein level of MKK1-nYFP, MKK2-nYFP and βC1-cYFP in the BiFC assay were shown by immunoblotting using anti-HA antibody. Combinations of agro-infiltrated constructs were indicated. Actin serves as a control. (JPG) [file ppat.1007728.s004.jpg]

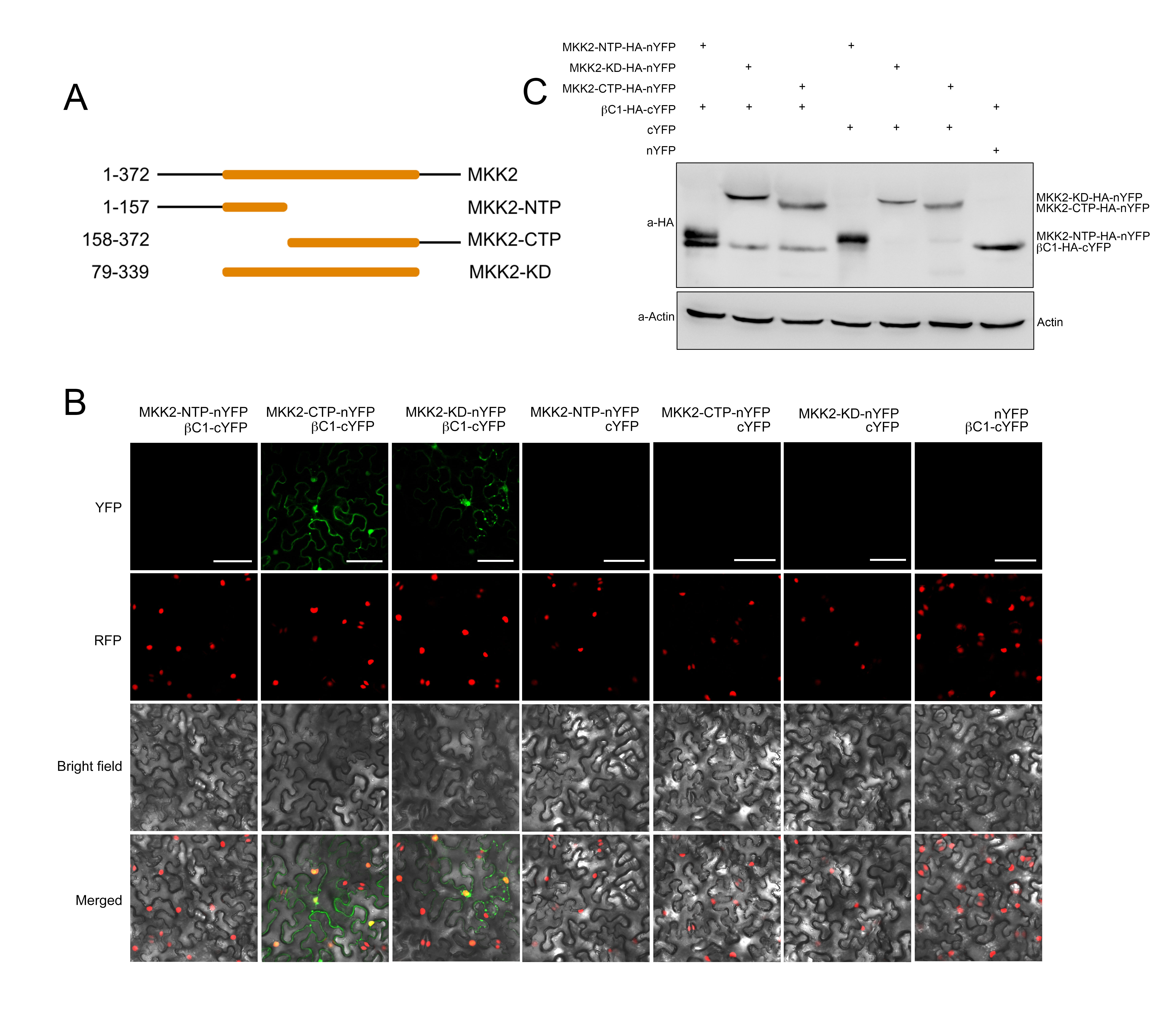

Supplement: S2 Fig — (A) Diagram of MKK2 truncated or deletion variants. (B) BiFC visualization of interaction between MKK2 mutants and βC1 in 35S-RFP-H2B transgenic N. benthamiana leaves. Combinations of the infiltrated constructs were indicated. Columns from left to right represent fluorescence of YFP, and RFP fluorescence, bright field and YFP/RFP/bright field overlay, respectively. Bars represent 50 μm. (C) The protein level of MKK2-NTP-nYFP, MKK2-CTP-nYFP, MKK2-KD-nYFP and βC1-cYFP in the BiFC assay were shown by immunoblotting using anti-HA antibody. Combinations of agro-infiltrated constructs were indicated. Actin serves as a control. (JPG) [file ppat.1007728.s005.jpg]

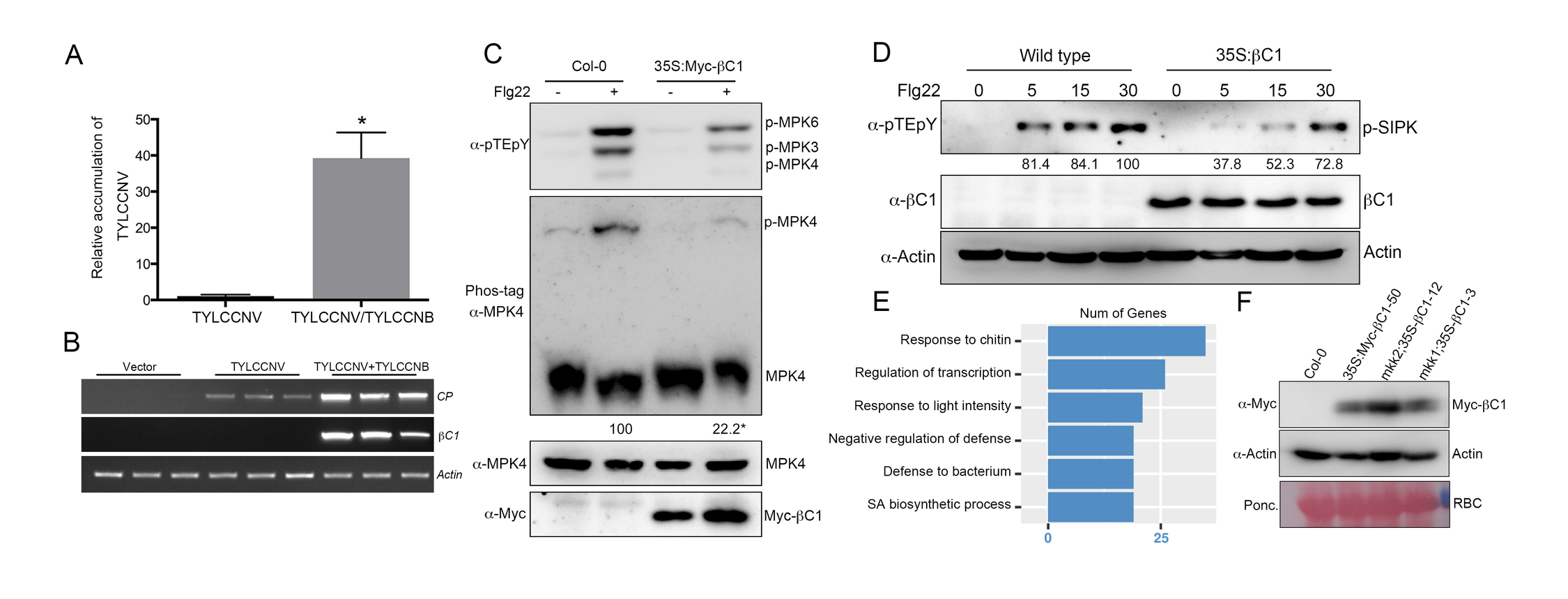

Supplement: S3 Fig — (A) Viral accumulation was determined by qPCR. The values represent viral DNA accumulation relative to level in TYLCCNV infected plants. The data are shown as means and SEM of three biological replicates. Asterisk indicates significant difference (p<0.05, Student’s t test). (B) The mRNA level of CP and βC1 were shown by RT-PCR, Actin serves as a control. (C) Flg22-induced MPK4 activation in Wild type and 35S-βC1 Arabidopsis. 10-day seedlings were treated with 100 nM flg22 for 15 min and subjected to immunoblot assays with an anti-pTEpY, anti-MPK4 or anti-Myc antibody. In phosphoaffinity-based SDS-PAGE, total protein were separated in a 10% SDS-PAGE gel supplemented with 50 mM phos-tag (Wako chemicals USA, Inc.), and MPK4 and p-MPK4 protein were detected with anti-MPK4 antibody. Three biological replicates were performed. Numbers indicate the average amount of phosphorylated MPK4 protein, and the values were normalized to flg22 treated wild-type samples. Asterisk indicates significant differences (p<0.05, Student’s t test). (D) Flg22-induced MAPK activation in Wild type and 35S-βC1 transgenic N. benthamiana. 8-day seedlings were treated with 100 nM flg22 for indicated time period and subjected to immunoblot assays with an anti-pTEpY, anti-βC1 or anti-Actin antibody. Accumulated phosphorylated NbSIPK level for three biological replicates was calculated and is shown below. (E) GO enrichmentanalysis of genes that were either significant up/down regulated in wild-type but not in 35S-Myc-βC1 or significant up/down regulated in 35S-Myc-βC1 but had a 1.5 times lower fold change than wild-type. (F) Amount of Myc-βC1 protein in wild type, mkk1 and mkk2 background. Total protein of 10-day T2 homozygous transgenic seedlings was subjected to immunoblot assays with an anti-Actin or anti-Myc antibody. Ponceau S staining of Rubisco (RBC) shows protein loading. (TIF) [file ppat.1007728.s006.tif]

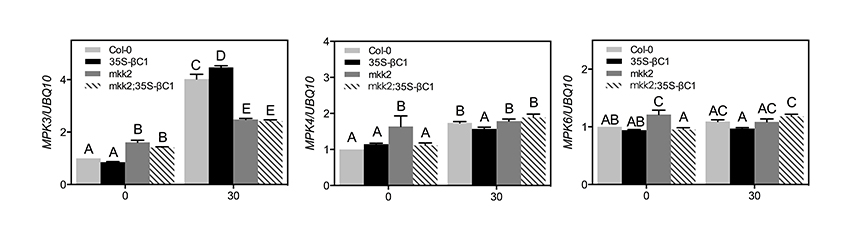

Supplement: S4 Fig — RT-qPCR analysis of MPK3, MPK4 and MPK6 in Col-0 and 35S-Myc-βC1 with or without flg22 treatment for 30 min. The letters indicate significant differences with a Student’s t test (P < 0.05). (JPG) [file ppat.1007728.s007.jpg]

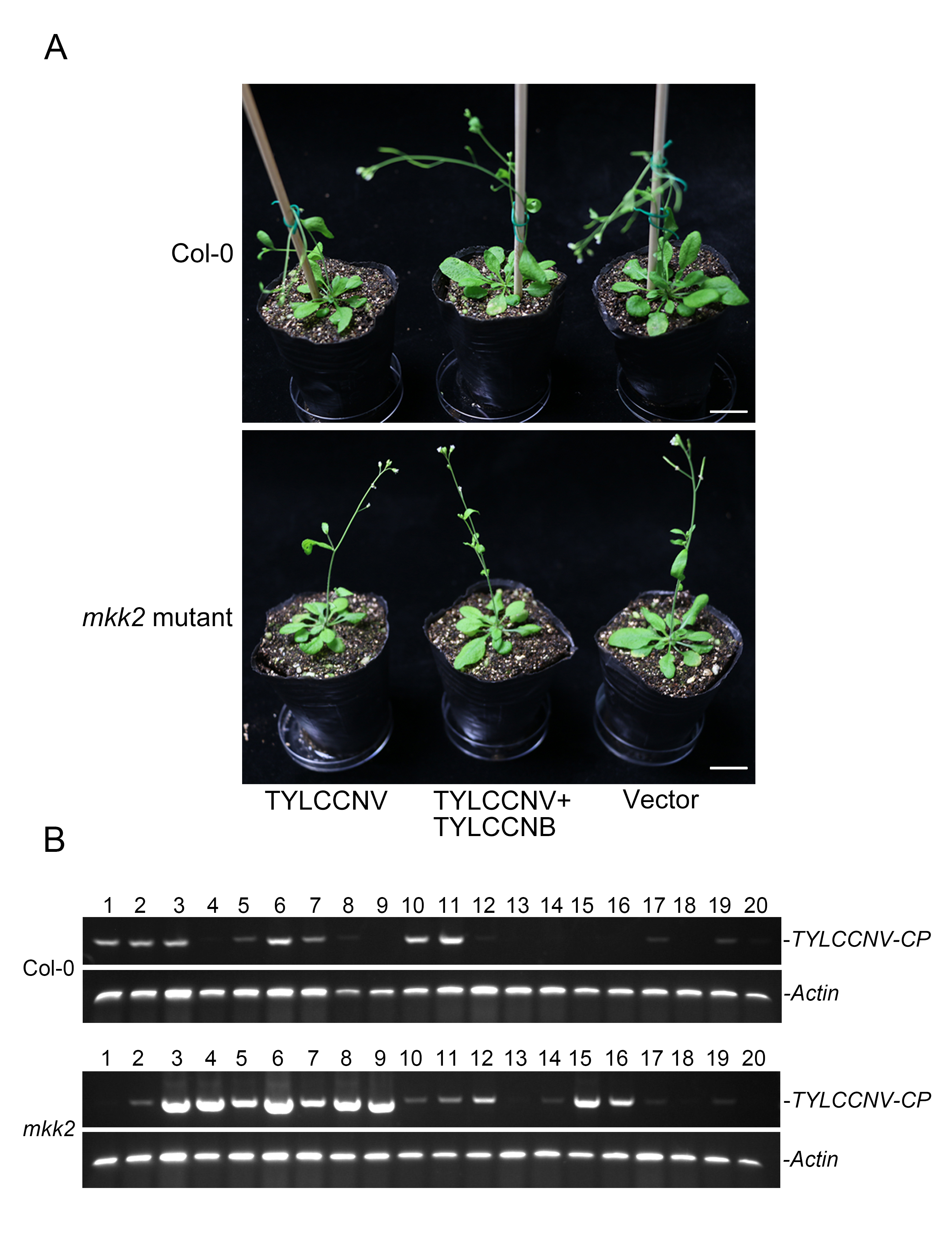

Supplement: S5 Fig — (A) TYLCCNV infected Arabidopsis did not exhibit developmental defect. Eight-leaf-period Arabdopsis seedlings were inoculated with A. tumefaciens harboring TYLCCNV/TYLCCNB, TYLCCNV infectious clone or empty vector, respectively. Phenotype was monitored 8 days post infiltration. Bars represent 2cm. (B) Virus CP gene of 20 TYLCCNV+TYLCCNB inoculated wild type or mkk2 mutant Arabidopsis plants was analyzed by PCR. Actin serves as a loading control. (JPG) [file ppat.1007728.s008.jpg]

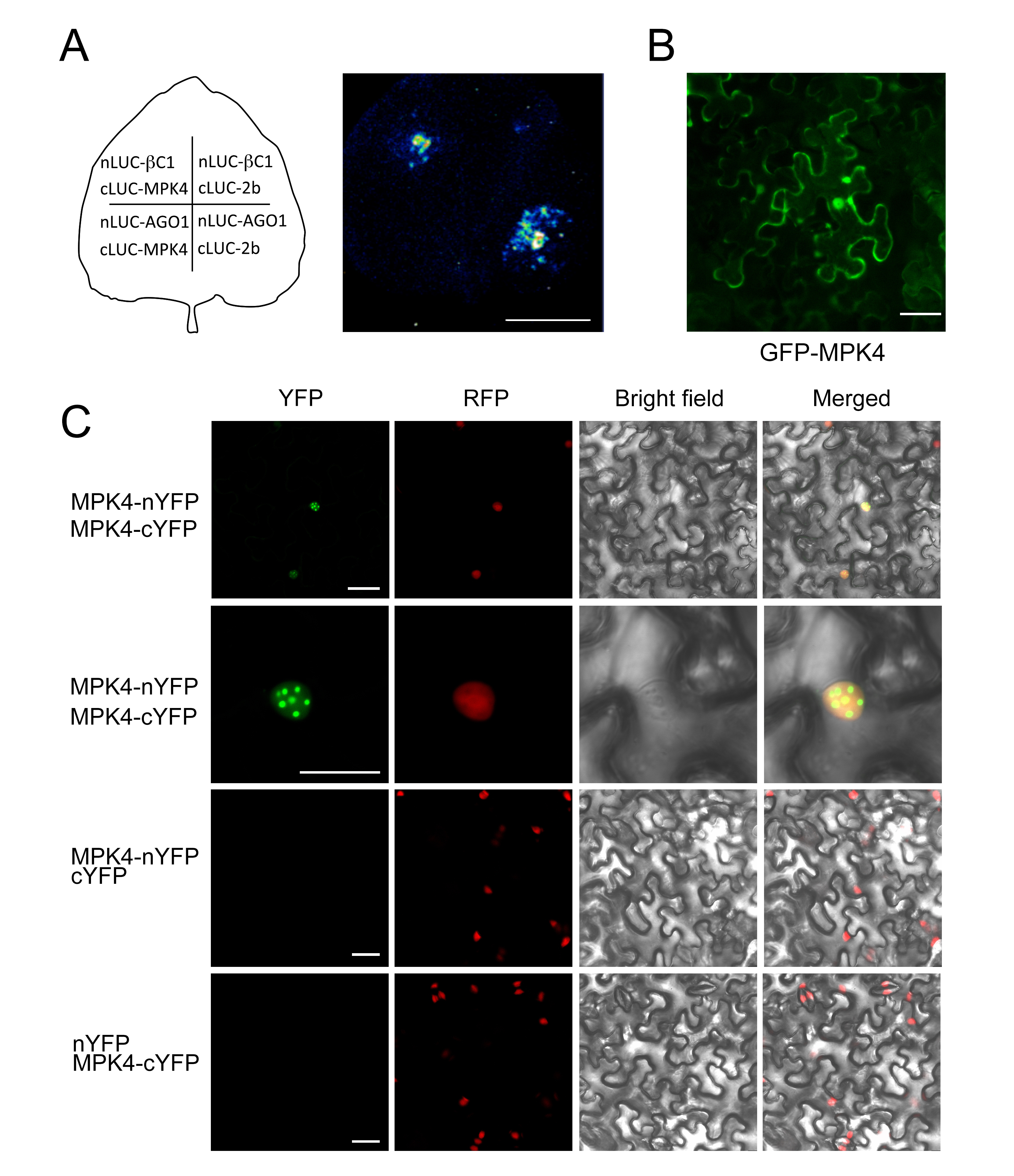

Supplement: S6 Fig — (A) LCI assay shows that βC1 interacts with MPK4 in planta. Different Combinations of NLuc and CLuc derivative constructs were co-infiltrated into N. benthamiana leaves for LCI assay. Infiltrated positions on the leaf were shown in the left panel. Fluorescence signal represents protein-protein interaction. Bar represents 5cm. (B) A. tumefaciens harboring GFP-MPK4 was infiltrated into N. benthamiana GFP fluorescence was analyzed using confocal microscopy. Bars represent 50 μm. (C) A. tumefaciens harboring combinations of indicated constructs were infiltrated into RFP-H2B transgenic N. benthamiana leaves. YFP or RFP fluorescence was analyzed using confocal microscopy. Columns from left to right represent YFP fluorescence, RFP fluorescence, bright field and YFP/RFP/bright field overlay. Bars represent 50 μm. (JPG) [file ppat.1007728.s009.jpg]

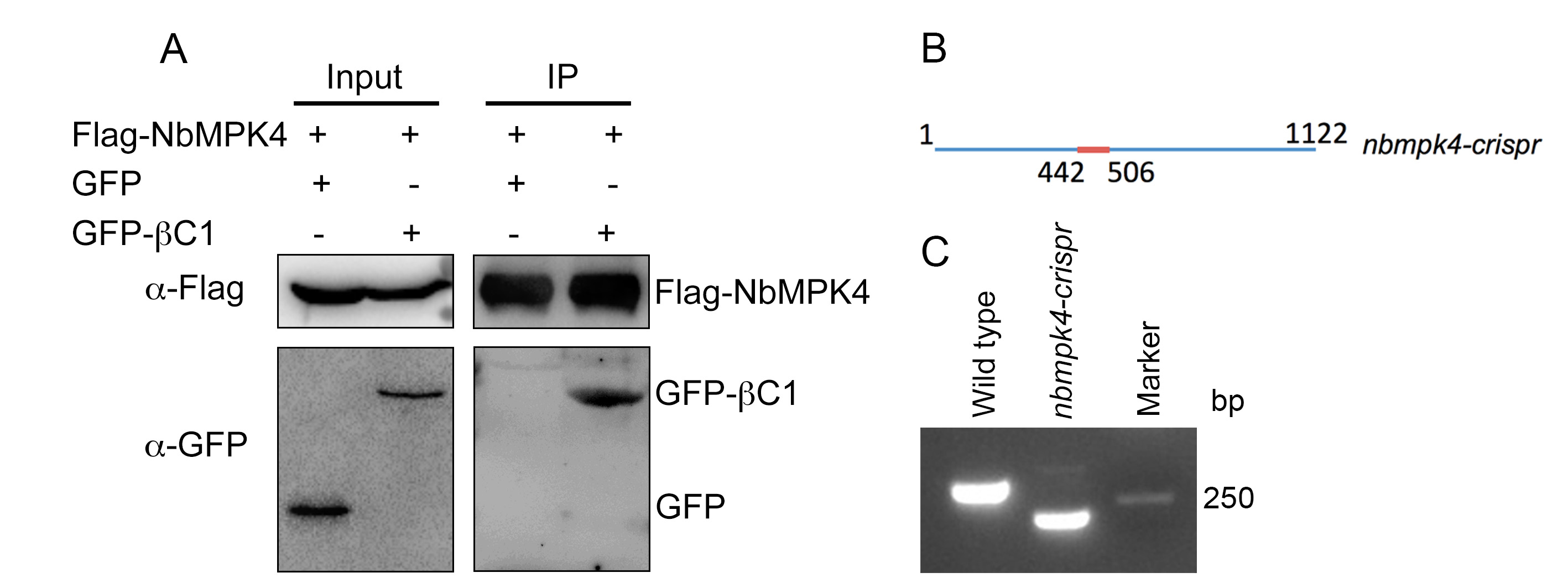

Supplement: S7 Fig — (A) Confirm the interaction between βC1 and NbMPK4 by Co-IP assay. N. benthamiana leaves were infiltrated with A. tumefaciens cells harboring 3Flag-NbMPK4 with GFP-βC1 or GFP for Co-IP assay. Samples were analyzed by immunoblot using anti-GFP and anti-Flag antibody (B) Location of single guide RNA target in NbMPK4 locus. 65 nucleotides were deleted in the exon of NbMPK4. (C) PCR analysis of a 284 nt long sequence which includes single guide RNA target region in NbMPK4 locus. (JPG) [file ppat.1007728.s010.jpg]
